# Burden and factors associated with schistosomiasis and soil-transmitted helminth infections among school-age children in Huambo, Uige and Zaire provinces, Angola

**DOI:** 10.1186/s40249-022-00975-z

**Published:** 2022-06-25

**Authors:** Adam W. Bartlett, Jose C. Sousa-Figueiredo, Roelofje C. van Goor, Paul Monaghan, Warren Lancaster, Rukaaka Mugizi, Elsa P. Mendes, Susana Vaz Nery, Sergio Lopes

**Affiliations:** 1 Global Health Program, Kirby Institute, University of New South Wales, New South Wales, Australia; 2 Department of Life Sciences, Natural History Museum, Wolfson Wellcome Biomedical Laboratories, London, UK; 3 Health Research Center Angola, Hospital Provincial, Bengo, Angola; 4 The Mentor Initiative, Luanda, Angola; 5 The END Fund, New York, NY USA; 6 Section for Control of Neglected Tropical Diseases, Department of Disease Control, National Directorate of Public Health, Ministry of Health, Luanda, Angola; 7 The Mentor Initiative, 4Th Floor (South Suite), Burns House, Harlands Road, Haywards Heath, R16 1PG UK

**Keywords:** Schistosomiasis, Soil-transmitted helminths, Circulating cathodic antigen, Rapid diagnostic test, Water, sanitation and hygiene

## Abstract

**Background:**

Schistosomiasis and soil-transmitted helminths (STHs) contribute high disease burdens amongst the neglected tropical diseases (NTDs) and are public health problems in Angola. This study reports the prevalence, intensity and risk factors for schistosomiasis and STH infection in Huambo, Uige and Zaire provinces, Angola, to inform a school-based preventive chemotherapy program.

**Methods:**

A two-stage cluster design was used to select schools and schoolchildren to participate in parasitological and water, sanitation and hygiene (WASH) surveys across Huambo, Uige, and Zaire provinces. Point-of-care circulating cathodic antigen and urinalysis rapid diagnostic tests (RDTs) were used to determine the prevalence of *Schistosoma mansoni* and *S. haematobium*, respectively. Kato-Katz was used to identify and quantify STH species and quantify and compare with RDTs for *S. mansoni*. Urine filtration was used to quantify and compare with RDTs for *S. haematobium*. Descriptive statistics were used for prevalence and infection intensity of schistosomiasis and STH infection. Performance of RDTs was assessed through specificity and Cohen’s Kappa agreement with microscopy. A multivariate regression analysis was used to determine demographic and WASH factors associated with schistosomiasis and STH infection.

**Results:**

A total 575 schools and 17,093 schoolchildren participated in the schistosomiasis survey, of which 121 schools and 3649 schoolchildren participated in the STH survey. Overall prevalence of *S. mansoni* was 21.2% (municipality range 0.9–74.8%) and *S. haematobium* 13.6% (range 0–31.2%), with an overall prevalence of schistosomiasis of 31.4% (range 5.9–77.3%). Overall prevalence of *Ascaris lumbricoides* was 25.1% (range 0–89.7%), hookworm 5.2% (range 0–42.6%), and *Trichuris trichiura* 3.6% (range 0–24.2%), with an overall prevalence of STH infection of 29.5% (range 0.8–89.7%). Ecological zone and ethnicity were factors associated with schistosomiasis and STH infection, with older age and female sex additional risk factors for *S. haematobium*.

**Conclusions:**

Most municipalities met World Health Organization defined prevalence thresholds for a schistosomiasis preventive chemotherapy program. A STH preventive chemotherapy program is indicated for nearly all municipalities in Uige and select municipalities in Huambo and Zaire. The association between ecological zone and ethnicity with schistosomiasis and STH infection necessitates further evaluation of home and school environmental, sociodemographic and behavioural factors to inform targeted control strategies to complement preventive chemotherapy programs.

**Supplementary Information:**

The online version contains supplementary material available at 10.1186/s40249-022-00975-z.

## Background

Schistosomiasis (infection caused by *Schistosoma* species) and soil-transmitted helminth (STH) infections including hookworms (*Necator americanus*, *Ancylostoma duodenale*, *Ancylostoma ceylanicum*), *Ascaris lumbricoides*, and *Trichuris trichiura* are prevalent in tropical and subtropical areas, particularly in low-resourced communities with limited access to safe water sources, sanitation and hygiene [1–3]. They cause a range of gastrointestinal and urogenital disease, that can lead to anaemia and malnutrition, and occasionally death [3]. The intensity of schistosomiasis or STH infection, which is typically measured by the number of eggs detected in stool or urine via microscopy (e.g., Kato Katz or urine filtration, respectively), is a key parasitological marker for estimating the disease burden associated with these infections [4–6]. School-age children are particularly susceptible to these infections as they are frequently exposed to contaminated soil and water (through playing, washing, eating and drinking) with less awareness of sanitation and hygiene, during an important period of physical and cognitive development [3]. Globally, STH infections contribute the greatest burden of disease of the neglected tropical diseases (NTDs), accounting for over 3.3 million disability-adjusted life years (DALYs) [7]. Schistosomiasis accounts for over 1.8 million DALYs, the third highest global burden attributable to a NTD behind STH infections and dengue [7]. As such, the World Health Organization (WHO) have targeted schistosomiasis and STH infections for elimination as a public health problem by 2030 [8]. For schistosomiasis, this means reducing the proportion of heavy intensity infection to < 1% in school-age children; while for STH infection, this means decreasing the proportion of moderate and heavy intensity infection to < 2% in school-age children [8–10].

An integral component to schistosomiasis and STH control programs is the implementation of large-scale preventive chemotherapy programs, consisting of regular distribution of anthelminthic therapy to entire populations or targeted to at-risk populations such as school-age children [3]. The WHO recommends the prevalence of schistosomiasis and STH infections as the main indicator for preventive chemotherapy programs and the frequency at which anthelminthic therapy is delivered [3, 11]. In addition to preventive chemotherapy, strategies to increase access to clean water, improved sanitation and adequate hygiene practices are recommended for sustainable control of schistosomiasis and STH infections [8].

Diagnostic techniques that facilitate efficient and cost-effective field work increase the capacity of field teams to survey larger cohorts with greater geographic spread, thereby sampling a more representative sample of the population being assessed for control measures. There has been evolving interest in the use of rapid diagnostic tests (RDTs) for schistosomiasis, with the first large-scale prevalence survey using RDTs to diagnose schistosomiasis undertaken in Namibia in 2012 and 2013 [12]. Using circulating cathodic antigen and Hemastix® to detect *S. mansoni* and *S. haematobium* respectively, this survey was able to survey 17,896 schoolchildren from 299 schools; and demonstrated a sensitivity above 80% and specificity above 95% for both RDTs when compared to Kato-Katz and urine filtration (the existing WHO reference techniques for *S. mansoni* and *S. haematobium* respectively) that were performed in a subset of children. Additional large-scale schistosomiasis RDT surveys have since been conducted that build upon the field work capacity and role of schistosomiasis RDTs in informing the implementation and impact of schistosomiasis control programs [13–17].

Schistosomiasis and STH infections are recognised public health problems in Angola [18, 19]. In 2005, United Nations Children’s Fund (UNICEF), WHO, Angolan Ministry of Health and other partners conducted a national prevalence survey of schistosomiasis and STH infection, which demonstrated a prevalence of intestinal parasites (STH species and *S. mansoni*) ranging from 26.5% to 75.9% and a prevalence of haematuria (as a proxy for *S. haematobium*) ranging from 11.8% to 40.6% across ecological zones (unpublished data). A cross-sectional survey in 2010 across three communes in Bengo province demonstrated the prevalence of at least one STH infection as 22.6% in preschool children and 31.6% in school-age children; and the prevalence of microhaematuria (as a proxy for *S. haematobium*) as 10.0% and 16.6% in preschool and school-age children respectively [18]. A cross-sectional survey (2013–2014) in children in Cubal estimated a crude prevalence of urinary schistosomiasis (by the presence of haematuria) of 61.2% [19]. Recognising the burden of schistosomiasis and STH infection, the Ministry of Health in Angola undertook a school-based preventive chemotherapy program with albendazole in 2013 followed by a school-based preventive chemotherapy program with praziquantel and albendazole from 2014. To inform the implementation of the program from 2014 onwards in each province, parasitological and water, sanitation and hygiene (WASH) surveys were carried out in stages across the country, with Huambo, Uige and Zaire the first three provinces to be surveyed. These provinces were the first to be surveyed as they were considered moderate- to high-risk areas for schistosomiasis and STH infections (unpublished data) with perennial and seasonal rivers, as well as having operational feasibility. This analysis reports the results from those surveys including: (i) the prevalence and intensity of schistosomiasis and STH infections; (ii) comparing the performance of RDTs and microscopy in diagnosing schistosomiasis; and (iii) demographic and WASH factors associated with schistosomiasis and STH infection.

## Methods

### Location

**Fig. 1:**
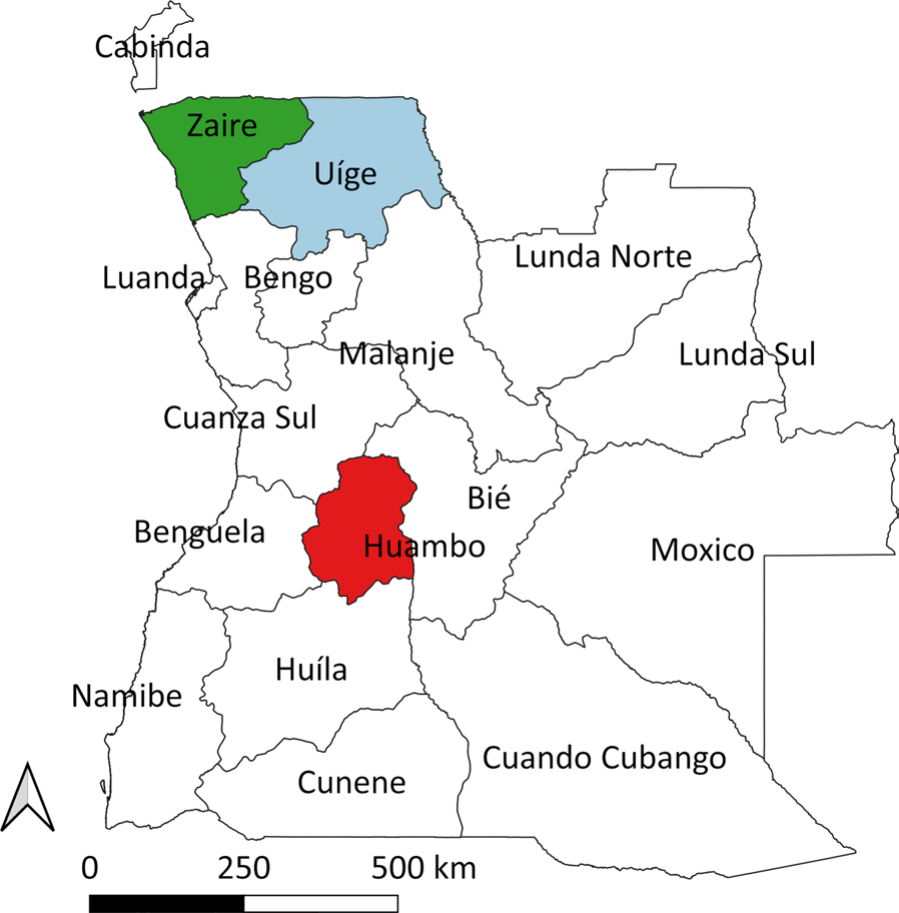
Location of Huambo, Uige and Zaire provinces in Angola

Figure 1 displays the location of Huambo, Uige and Zaire provinces in Angola, which is situated on the Western coast of Southern Africa. Angola is divided into six homogenous ecological zones, with the surveys being conducted in three of these ecological zones: Northern coastal, Coffee area and Central highland. The Northern coastal zone is primarily dense bush and savannah, with an average annual temperature of 25–26 °C, and an annual rainfall < 800 mm. The Coffee area zone consists of dense and humid forests, with an average annual temperature of 23–24 °C, and an annual rainfall of 1200–1500 mm. The Central highland zone has open forests and savannah, with an average annual temperature of 18–20 °C, and an annual rainfall of 1400 mm [20].

### Study design and sampling

A two-stage cluster design was employed to select schools and schoolchildren to participate in the surveys. The surveys consisted of: (i) a RDT survey to determine the prevalence of *S. mansoni* and *S. haematobium*; and (ii) a microscopy survey to detect and quantify STH infection (hookworm, *A. lumbricoides*, and *T. trichiura*), as well as quantify and provide diagnostic comparison with RDTs for *S. mansoni* and *S. haematobium*. The primary sampling frame was all of the registered primary and combined schools across Huambo, Uige and Zaire provinces. Following a similar approach to the schistosomiasis and STH mapping conducted in Namibia [12], sample size calculations for schistosomiasis prevalence were conducted using an estimated prevalence of 25%, precision of 5%, confidence level of 95% and design effect of 2.5 (using Epi Info v7.1.2; CDC Atlanta, USA). Based on these sample size calculations and the number of registered schools across Huambo, Uige and Zaire, a mapping resolution of 1 in 4 schools was deemed sufficient, resulting in 575 schools to be surveyed across the three provinces. The one in four schools for the schistosomiasis survey were selected using systematic random sampling and was adjusted to ensure representation from each municipality in each province. Of schools selected for the schistosomiasis survey, one in five were selected to participate in the STH survey. This sampling strategy resulted in 121 schools to be surveyed for STHs, with representation from each municipality, which provides a larger sample size than the WHO recommended five to ten schools for each ecological zone [3]. In selecting schoolchildren, a sampling frame was created of all children attending school on the day of the survey, from which 30 children (15 males and 15 females) were selected using systematic random sampling.

### Field work

Prior to field work commencing, meetings were held with representatives from the NTD program of the Ministry of Health, as well as with the respective provincial Public Health Departments. A week-long training workshop for field team members was conducted by JCSF that included information on clinical aspects and diagnosis of each infection, logistics, and data collection and management. Field teams then conducted pilot surveys over 3 days in schools in the provincial capitals to practice field procedures. Field teams collected data per province in three periods in 2014. Zaire province data collection took place between March 23 and April 2, Uige province data collection occurred between June 2 and July 18, and Huambo province data collection was undertaken between June 23 and August 12. To our knowledge, there were no specific health interventions implemented that would differentially affect the results obtained from the field work conducted across the different time periods in the three provinces. Figure 1 shows the location of Huambo, Uige and Zaire provinces in Angola.

### Survey procedures

Field work teams were designated to either perform schistosomiasis RDTs or perform microscopy to detect and quantify STH and *Schistosoma* species. These teams operated independently in the field but were coordinated for schools selected to participate in both the schistosomiasis and STH surveys. On the day of the survey, selected schools were visited by field teams to explain the surveys to the school director and obtain written consent for the school and the schoolchildren to participate. Each participating child was given a single sample collection vial for urine, and those participating in the STH survey were also provided with a single sample collection vial for stool. The samples were returned to the field workers and analysed on the same day. Upon receipt of samples, the age of each child was recorded, and all participating children were provided with one tablet of albendazole and the appropriate number of tablets of praziquantel, as determined using a dose pole. For the schistosomiasis survey, technicians used the point-of-care circulating cathodic antigen (POC-CCA®) (Rapid Medical Diagnostics, Pretoria, RSA) test to detect *S. mansoni* and Hemastix® (Bayer, UK) to detect haematuria as a proxy for *S. haematobium*. For the STH survey, microscopists used the Kato-Katz technique (single smear) to detect and quantify STH species (hookworm, *A. lumbricoides* and *T. trichiura*). Field teams performing microscopy also analysed specimens to detect and quantify *S. mansoni* and *S. haematobium* using Kato-Katz and the urine filtration technique respectively. A questionnaire was conducted in an interview with the school director of each school to evaluate: (i) the presence and functionality of latrines at the school; (ii) the accessibility to safe drinking water at the school; (iii) whether the schoolchildren had previously received preventive chemotherapy for STHs; and (iv) their own knowledge of schistosomiasis. Data were entered manually into data entry forms by the field workers. The data entry forms were collated then the data was manually entered into an electronic database.

### Detection and classification of infection

Single urine samples were collected from all schoolchildren to evaluate the prevalence of *S. mansoni* and *S. haematobium* using RDTs. The POC-CCA® results were graded as “negative”, “trace”, “+”, “++” and “+++” [21]. The Hemastix® results were graded as “negative”, “trace not haemolysed”, “trace haemolysed”, “+”, “++” and “+++” according to manufacturer’s instructions. Single stool samples were used for microscopy with the Kato-Katz technique to detect and determine the number of eggs per gram of faeces for STH species and *S. mansoni* [3]. The urine filtration technique was used to determine the number of *S. haematobium* eggs per 10 ml of urine [3].

### Ethics statement

Approval for the survey protocol was obtained by the Ministry of Public Health of Angola (101/GD/DNSP/2014). Informed written consent was obtained from the school directors of each school to allow field teams to visit, then select and invite schoolchildren to participate in the school-based surveys, in agreement with the consenting process approved by the Ministry of Public Health of Angola.

### Statistical analysis

Descriptive statistics were used to report the characteristics of the schools and schoolchildren that participated in the schistosomiasis and STH surveys for each municipality across Huambo, Uige and Zaire provinces. Prevalence, with 95% confidence intervals (*CI*s), for schistosomiasis and STH infection was calculated for each municipality with adjustments made for the cluster survey design. Prevalence was separately determined for when RDT trace readings were considered positive and for when they were considered negative. Descriptive statistics were used to report infection intensity for STH and *Schistosoma* species as determined by microscopy. The spatial distribution of schistosomiasis and STH prevalence was represented graphically using QGIS version 3.18 (QGIS.org, 2022. QGIS Geographic Information System. QGIS Association. http://www.qgis.org). Diagnostic agreement between the RDTs (for trace readings as positive and trace readings as negative) and microscopy was performed using Cohen’s Kappa agreement statistics (very good, κ > 0.8; good, 0.6 < κ ≤ 0.8; moderate, 0.4 < κ ≤ 0.6; fair, 0.2 < κ ≤ 0.4; poor, κ ≤ 0.2). The specificity of the RDTs in detecting *S. mansoni* and *S. haematobium* was calculated using Kato-Katz and urine filtration as the comparator, respectively. A logistic regression analysis was used, using a mixed-effects model and adjusted for the cluster survey design, to assess factors associated with specific *Schistosoma* or STH species, as well as any schistosomiasis or STH infection. Covariates with a *P*-value < 0.2 on univariate logistic regression analysis were included in a multivariate logistic regression analysis. The multivariate logistic regression analysis was conducted to determine adjusted odds ratios (a*OR*s) in a stepwise fashion maintaining covariates that retained a *P*-value < 0.05. Covariates included sex, age, school setting (rural, urban), ecological zone (Northern coastal, Coffee area, Central highland), ethnicity (Kikongo, Kimbundo, Umbundo), previous school deworming in 2013, presence of functional latrines at school, and presence of a reliable safe drinking water source at school. The primary logistic regression analysis was conducted considering infection determined by RDT trace readings as positive, with a secondary logistic regression analysis performed considering RDT trace readings as negative. All statistical analyses were performed using Stata version 17.0 (StataCorp LP, College Station, Texas).

## Results

### Survey population

There were a total 575 schools across Huambo, Uige and Zaire provinces from which 17,093 schoolchildren participated in the schistosomiasis survey. From this, 121 schools and 3649 schoolchildren also participated in the STH survey. Overall, there was equal representation of males and females, and the median age of participants was 11 [interquartile range (*IQR*): 10–13] years. Table 1 shows the demographics of the schistosomiasis and STH survey populations for each province, and Additional file 1: Table S1 provides the demographics of the schistosomiasis and STH surveys for each municipality.

**Table 1:** Demographics of participants in the schistosomiasis and soil-transmitted helminth surveys for Huambo, Uige and Zaire provinces

	Huambo		Uige		Zaire		Total	
	RDT *N* = 7620	Microscopy *N* = 1501	RDT *N* = 7793	Microscopy *N* = 1818	RDT *N* = 1680	Microscopy *N* = 330	RDT *N* = 17,093	Microscopy *N* = 3649
Schools	254	50	265	60	56	11	575	121
Students								
Male	3796 (49.2%)	750 (50.0%)	3916 (50.3%)	903 (49.7%)	838 (49.9%)	165 (50.0%)	8550 (50.0%)	1818 (49.8%)
Female	3824 (50.2%)	751 (50.0%)	3877 (49.8%)	915 (50.3%)	842 (50.1%)	165 (50.0%)	8543 (50.0%)	1831 (50.2%)
Age, years (*IQR*)	11 (10–13)	11 (10–13)	11 (10–13)	11 (9–13)	12 (10–13)	12 (10–14)	11 (10–13)	11 (10–13)
Setting								
Rural	6150 (80.7%)	1411 (94.0%)	6740 (86.6%)	1643 (90.5%)	1350 (80.4%)	270 (81.8%)	14,249 (83.4%)	3324 (91.1%)
Urban	1470 (19.3%)	90 (6.0%)	1044 (13.4%)	173 (9.5%)	330 (19.6%)	60 (18.2%)	2844 (16.6%)	323 (8.9%)
Ethnicity								
Kikongo	0	0	6257 (80.3%)	1488 (81.9%)	1680 (100%)	330 (100%)	7937 (46.4%)	1818 (49.9%)
Kimbundo	0	0	1536 (19.7%)	328 (18.1%)	0	0	1536 (9.0%)	328 (9.0%)
Umbundo	7620 (100%)	1501 (100%)	0	0	0	0	7620 (44.6%)	1501 (41.2%)
Ecological zone								
Northern coastal	0	0	0	0	930 (55.4%)	210 (63.6%)	930 (5.4%)	210 (5.8%)
Coffee area	0	0	2553 (32.8%)	738 (40.6%)	750 (44.6%)	120 (36.4%)	3303 (19.3%)	858 (23.5%)
Central highland	7620 (100%)	1501 (100%)	5240 (67.2%)	1078 (59.4%)	0	0	12,860 (75.2%)	2579 (70.7%)

*N* number of school children participating in the respective surveys, *IQR* interquartile range, *RDT* rapid diagnostic test

### Prevalence and infection intensity

**Fig. 2:**
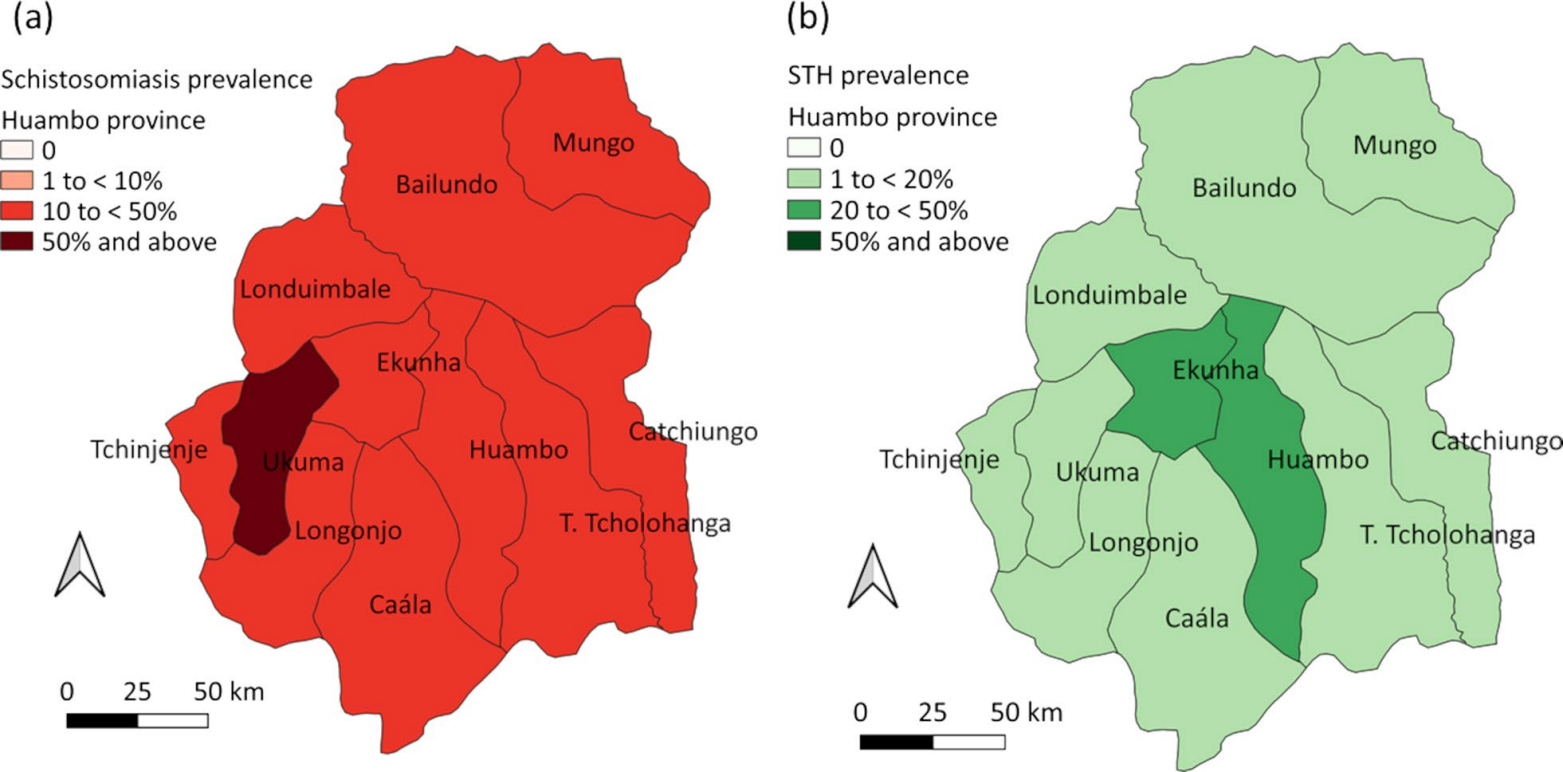
Prevalence of **a** schistosomiasis and **b** soil-transmitted helminth (STH) infections for Huambo province (based on rapid diagnostic tests, considering readings as positive)

**Fig. 3:**
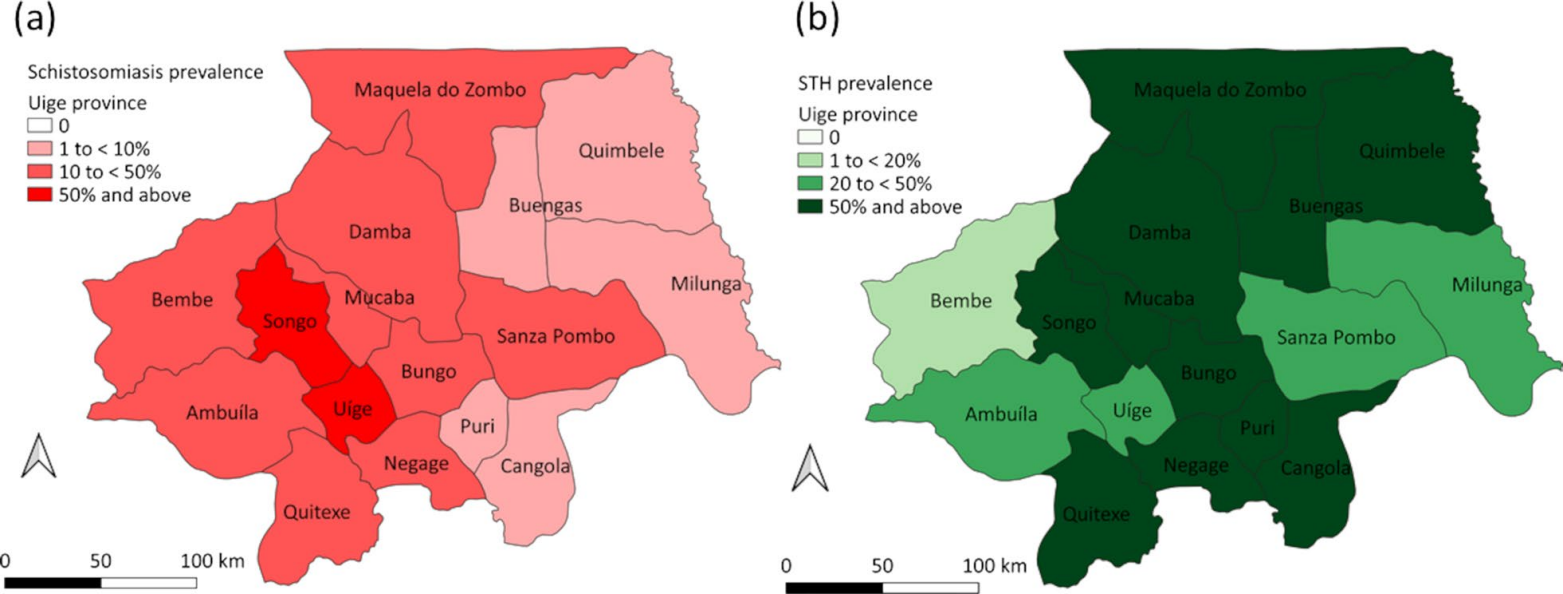
Prevalence of **a** schistosomiasis and **b** soil-transmitted helminth (STH) infections for Uige province (based on rapid diagnostic tests, considering readings as positive)

**Fig. 4:**
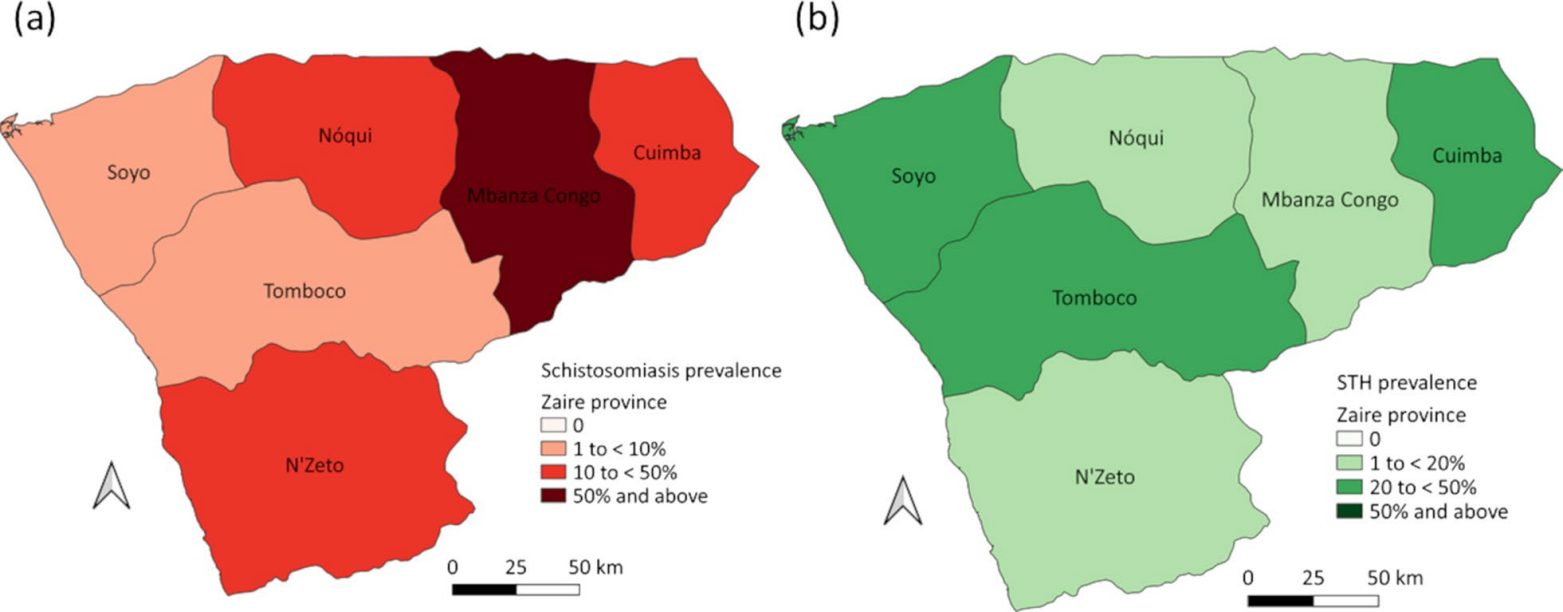
Prevalence of **a** schistosomiasis and **b** soil-transmitted helminth (STH) infections for Zaire province (based on rapid diagnostic tests, considering readings as positive)

When considering RDT trace readings as positive, the overall prevalence (adjusted for clustering) of *S. mansoni* was 21.2% (95% *CI*: 18.1–24.6) and *S. haematobium* was 13.6% (95% *CI*: 11.9–15.6), with an overall prevalence of any schistosomiasis of 31.4% (95% *CI*: 28.2–34.7) (Table 2). When considering RDT trace readings as negative, the overall prevalence (adjusted for clustering) of *S. mansoni* was 11.8% (95% *CI*: 9.7–14.3) and *S. haematobium* was 8.4% (95% *CI*: 7.1–9.9), with an overall prevalence of any schistosomiasis of 19.0% (95% *CI*: 16.7–21.5) (Table 2). Additional file 1: Table S2 provides the prevalence (adjusted for clustering) of schistosomiasis as determined by RDTs for each municipality. Figures 2a, 3a and 4a show the spatial distribution of schistosomiasis infection for each municipality (as determined when considering RDT trace readings as positive) across Huambo, Uige and Zaire provinces accordingly. Of the 3131 POC-CCA® results with a detectable reading 1406 (44.9%) were trace readings, 1052 (33.6%) were “+”, 400 (12.8%) were “++”, and 273 (8.7%) were “+++”. Of the 2005 Hemastix® results with a detectable reading, 767 (38.3%) were trace readings, 567 (28.3%) were “+”, 299 (14.9%) were “++”, and 372 (18.6%) were “+++”.

**Table 2:** Prevalence of schistosomiasis based on rapid diagnostic tests and microscopy for Huambo, Uige and Zaire provinces

	Huambo	Uige	Zaire	Total
*S. mansoni*				
RDT	*N* = 7620	*N* = 7793	*N* = 1680	*N* = 17,093
Overall when trace positive, % (range)	20.8 (6.6–47.2)	21.2 (2.0–74.8)	23.1 (0.9–47.1)	21.2 (0.9–74.8)
Overall when trace negative, % (range)	8.9 (2.7–18.4)	16.1 (0.9–55.0)	14.1 (0–29.9)	11.8 (0–55.0)
Microscopy	*N* = 1501	*N* = 1818	*N* = 330	*N* = 3649
Light intensity, % (95% *CI*)	0.2 (0.05–0.9)	20.0 (9.0–38.6)	0.1 (0.01–1.1)	8.9 (3.5–20.4)
Moderate intensity, % (95% *CI*)	0	0.04 (0.01–0.3)	0	0.02 (0.003–0.1)
Heavy intensity, % (95% *CI*)	0	0	0	0
Overall, % (range)	0.2 (0–0.8)	20.0 (0–87.4)	0.1 (0–1.3)	8.9 (0–87.4)
*S. haematobium*				
RDT	*N* = 7620	*N* = 7793	*N* = 1680	*N* = 17,093
Overall when trace positive, % (range)	18.7 (0–29.8)	5.6 (0.7–9.9)	11.3 (3.8–31.2)	13.6 (0–31.2)
Overall when trace negative, % (range)	11.9 (0–20.8)	2.8 (0.3–9.6)	7.0 (0–18.0)	8.4 (0–20.8)
Microscopy	*N* = 1500	*N* = 1615	*N* = 330	*N* = 3445
Light intensity, % (95% *CI*)	5.7 (3.3–9.6)	0.6 (0.2–1.7)	1.7 (0.3–8.7)	3.4 (2.1–5.4)
Heavy intensity, % (95% *CI*)	2.7 (1.3–5.5)	0.1 (0.05–0.4)	1.6 (0.2–12.7)	1.6 (0.8–3.1)
Overall, % (range)	8.4 (0–34.4)	0.7 (0–5.9)	3.3 (0–6.6)	5.0 (0–34.4)
Any schistosomiasis				
RDT	*N* = 7620	*N* = 7793	*N* = 1680	*N* = 17,093
Overall when trace positive, % (range)	34.7 (26.9–57.0)	25.3 (5.9–77.3)	32.2 (6.9–51.2)	31.4 (5.9–77.3)
Overall when trace negative, % (range)	19.1 (12.5–31.5)	18.4 (3.5–55.6)	20.1 (1.6–34.1)	19.0 (1.6–55.6)
Microscopy	*N* = 1501	*N* = 1818	*N* = 330	*N* = 3649
Overall, % (range)	8.6 (0–34.4)	24.7 (0–87.4)	3.3 (0–6.6)	14.5 (0–87.4)

*N* number of school children providing specimens for the respective surveys, *Range* municipality range, *RDT* rapid diagnostic test. Adjusted for clustering at school level

For the subset of schoolchildren who submitted a sample for microscopy the overall prevalence (adjusted for clustering) of *S. mansoni* was 8.9% (95% *CI*: 3.6–20.5) and *S. haematobium* was 5.0% (95% *CI*: 3.1–8.0), with an overall prevalence of any schistosomiasis of 14.5% (95% *CI*: 8.2–24.4) (Additional file 1: Table S2). Nearly all *S. mansoni* infections were of light intensity (183/184, 98.5%), while for *S. haematobium* 62.2% (145/233) were light and 37.8% (88/233) were heavy intensity infections. The overall prevalence (adjusted for clustering) of moderate or heavy intensity *S. mansoni* infection was 0.2% (95% CI 0.003–0.1), and the overall prevalence (adjusted for clustering) of heavy intensity *S. haematobium* infection was 1.6% (95% *CI*: 0.8–3.1) (Table 2).

The overall prevalence (adjusted for clustering) of any STH infection across the three provinces was 29.5% (95% *CI*: 23.2–36.6) (Table 3). *Ascaris lumbricoides* was the most common STH species, with an overall prevalence of 25.1% (95% *CI*: 19.4–31.9) (Table 3). The overall prevalence of hookworm was 5.2% (95%* CI*: 3.7–7.3) and the overall prevalence of *T. trichiura* was 3.6% (95% *CI*: 2.6–5.0) (Table 3). Additional File 1: Table S3 shows the prevalence of STH species for each municipality. Figures 2b, 3b and 4b display the spatial representation of STH prevalence for each municipality across Huambo, Uige and Zaire provinces respectively. Nearly all STH infections were of light intensity, with 100% (324/324) hookworm, 98.9% (1114/1126) *A. lumbricoides*, and 98.2% (167/170) *T. trichiura* infections determined light intensity. The overall prevalence (adjusted for clustering) of moderate or heavy intensity infections was 0.7% (95% *CI*: 0.2–2.4) for *A. lumbricoides*, 0.04% (95% *CI*: 0.01–0.2) for *T. trichiura* and 0% for hookworm (Table 3).

**Table 3:** Prevalence of soil-transmitted helminths by microscopy for Huambo, Uige and Zaire provinces

	Huambo	Uige	Zaire	Total
Hookworm	*N* = 1501	*N* = 1816	*N* = 330	*N* = 3647
Light intensity, % (95% *CI*)	0.1 (0.02–1.1)	11.1 (7.2–16.8)	4.2 (0.9–18.1)	5.2 (3.7–7.3)
Moderate intensity, % (95% *CI*)	0	0	0	0
Heavy intensity, % (95% *CI*)	0	0	0	0
Overall, % (range)	0.1 (0–0.6)	11.1 (0–42.6)	4.2 (0–8.0)	5.2 (0–42.6)
*A. lumbricoides*	*N* = 1501	*N* = 1816	*N* = 330	*N* = 3647
Light intensity, % (95% *CI*)	12.4 (6.6–21.9)	39.4 (30.1–49.6)	16.5 (8.7–28.9)	24.5 (19.1–30.8)
Moderate intensity, % (95% *CI*)	(0.02–0.9)	1.4 (0.4–5.0)	0	0.7 (0.2–2.4)
Heavy intensity, % (95% *CI*)	0	0	0	0
Overall, % (range)	12.5 (0–32.8)	40.8 (3.4–89.7)	16.5 (0–30.9)	25.1 (0–89.7)
*T. trichiura*	*N* = 1496	*N* = 1813	*N* = 330	*N* = 3639
Light intensity, % (95% *CI*)	0.6 (0.2–1.4)	7.1 (5.2–9.7)	2.4 (0.8–6.7)	3.6 (2.5–5.0)
Moderate intensity, % (95% *CI*)	0.08 (0.02–0.4)	0	0	0.04 (0.01–0.2)
Heavy intensity, % (95% *CI*)	0	0	0	0
Overall, % (95% *CI*)	0.7 (0–4.2)	7.1 (0–24.2)	2.4 (0–7.4)	3.6 (0–7.4)
Any STH	*N* = 1501	*N* = 1818	*N* = 330	*N* = 3647
Overall, % (range)	13.1 (0.8–33.2)	49.4 (5.2–89.7)	20.6 (6.7–36.8)	29.5 (0.8–89.7)

*CI* confidence interval, *N* number of school children providing specimens, *Range* municipality range, *STH* soil-transmitted helminth. Adjusted for clustering at school level

### Diagnostic performance of RDTs compared to microscopy

Table 4 summarises the comparative diagnostic performance between schistosomiasis RDTs and microscopy. When considering RDT trace readings as positive, the diagnostic agreement between POC-CCA® and Kato-Katz in detecting *S. mansoni* infection was fair (κ = 0.38, *P* < 0.001), and the diagnostic agreement between Hemastix® and urine filtration was moderate (κ = 0.51, *P* < 0.001). The specificity of the RDTs compared to microscopy was 88.8% (95% *CI*: 87.7–89.9) for POC-CCA® and 92.7% (95% *CI*: 91.7–93.5) for Hemastix®. When considering RDT trace readings as negative, the diagnostic agreement between POC-CCA® and Kato-Katz was moderate (κ = 0.57, *P* < 0.001), and the diagnostic agreement between Hemastix® and urine filtration was moderate (κ = 0.53, *P* < 0.001). The specificity of the RDTs compared to microscopy was 95.2% (95% *CI*: 94.4–95.9) for POC-CCA® and 94.9% (95%* CI*: 94.1–95.6) for Hemastix®.

**Table 4:** Diagnostic performance between schistosomiasis rapid diagnostic tests and microscopy

	RDT (trace positive)					RDT (trace negative)				
	Detected	Not detected	Agreement	Kappa statistic^a^	*P*-value	Detected	Not detected	Agreement	Kappa statistic^a^	*P*-value
*S. mansoni*										
Kato-Katz										
Detected	155	29	88.6%	0.38	< 0.001	150	34	94.5%	0.57	< 0.001
Not detected	387	3078				167	3298			
*S. haematobium*										
Urine filtration										
Detected	181	52	91.6%	0.51	< 0.001	157	76	93.0%	0.53	< 0.001
Not detected	236	2976				164	3048			

^a^Kappa agreement classification: ≤ 0.20 = poor; 0.21–0.40 = fair; 0.41–0.60 = moderate; 0.61–0.80 = good; 0.81–1.00 = very good

*RDT* rapid diagnostic test

There were 225 samples that had a trace POC-CCA® reading and were analysed by Kato-Katz for *S. mansoni* (Additional file 1: Table S4). Of these, 97.8% (220/225) were negative on microscopy and 2.2% (5/225) were determined light intensity *S. mansoni* infection, with no moderate or heavy intensity *S. mansoni* infections identified. For *S. haematobium*, there were 96 samples that had a trace Hemastix® reading and were analysed by urine filtration (Additional file 1: Table S4). Of these 75.0% (72/96) were negative on microscopy, 18.8% (18/96) were determined light intensity infection and 6.2% (6/96) were determined heavy intensity infection.

### School questionnaire on school WASH indicators

Table 5 summarises the results of the school WASH questionnaire for each province. Of the 575 schools participating in the parasitological surveys there were 217 (37.7%) schools that had functional bathrooms and 134 (23.3%) that had a reliable safe drinking water source. There were 81.9% (472/575) that received school-based anthelminthic preventive chemotherapy in 2013. Additional file 1: Table S5 displays results from the WASH questionnaire for all municipalities across Huambo, Uige and Zaire provinces.

**Table 5:** Results from school water, sanitation and hygiene questionnaires for Huambo, Uige and Zaire provinces

	Huambo *N* = 254 *n* (%)	Uige *N* = 265 *n* (%)	Zaire *N* = 56 *n* (%)	Total *N* = 575 *n* (%)
How many schools had bathrooms?	148 (58.3)	127 (47.9)	40 (71.4)	315 (54.8)
How many schools had bathrooms in good condition?	124 (48.8)	81 (30.6)	12 (21.4)	217 (37.7)
How many schools had a reliable safe drinking water source?	86 (33.9)	40 (15.1)	8 (14.3)	134 (23.3)
Water source type?				
Tap	63 (24.8)	20 (7.5)	2 (3.6)	85 (14.8)
Hole/others	18 (7.1)	20 (7.5)	6 (10.7)	49 (8.5)
How many schools were dewormed in 2013?	203 (79.9)	218 (82.3)	51 (91.1)	472 (82.1)
School Directors’ demonstrated knowledge of schistosomiasis	81 (31.9)	55 (20.8)	29 (51.8)	165 (28.7)

*N*: number of school survey responses. 5 responses are missing for water source type in Huambo

### Factors associated with schistosomiasis or STH infection

On multivariate regression analysis, factors associated with *S. mansoni* included: ecological zone, with schoolchildren in the Central highland [a*OR* 2.90 (95% *CI*: 1.20–7.02), *P* = 0.02] or Coffee area [a*OR* 23.21 (95% *CI*: 9.13–58.98), *P* < 0.001] ecological zones more likely to be infected compared to schoolchildren in the Northern coastal ecological zone; and ethnicity, with schoolchildren of Kikongo [a*OR* 0.34 (95% *CI*: 0.24–0.49), *P* < 0.001] or Kimbundo [a*OR* 0.40 (95% *CI*: 0.21–0.77), *P* = 0.006] ethnicity less likely to be infected than schoolchildren of Umbundo ethnicity (Table 6). For *S. haematobium*, schoolchildren in the Coffee area [a*OR* 0.42 (95% *CI*: 0.19– 0.91), *P* = 0.03] or Central highland [a*OR* 0.29 (95% *CI*: 0.13–0.64), *P* = 0.002] ecological zones were less likely to be infected compared to schoolchildren in the Northern coastal ecological zone; as well as schoolchildren of Kikongo [a*OR* 0.21 (95% *CI*: 0.15–0.29), *P* < 0.001] or Kumbundo ethnicity [a*OR* 0.36 (95% *CI*: 0.27–0.49), *P* < 0.001] were less likely to be infected than schoolchildren of Umbundo ethnicity. Other factors associated with *S. haematobium* infection included a higher odds for females [a*OR* 1.51 (95% *CI*: 1.27–1.80), *P* < 0.001] compared to males, age more than 12 years compared to age less than 11 years [a*OR* 1.76 (95% *CI*: 1.40–2.22), *P* < 0.001], and attending school in an urban setting [a*OR* 1.60 (95% *CI*: 1.18–2.18), *P* = 0.003] compared to a rural setting (Table 6). Factors associated with any schistosomiasis infection are displayed in Table 6. Additional file 1: Table S6 shows the results of the multivariate regression analyses when considering schistosomiasis RDT trace readings as negative. Similar factors associated with any schistosomiasis infection, irrespective of whether RDT trace readings were considered positive or negative, included a higher odds of infection for age > 12 years (compared to age < 11 years), urban setting (compared to rural) and Coffee area ecological zone (compared to Northern coastal ecological zone), while a lower odds of infection were found for Kikongo or Kimbundo ethnicity compared to Umbundo ethnicity.

**Table 6:** Factors on multivariate analysis associated with schistosomiasis

	*S. mansoni*			*S. haematobium*			Any schistosomiasis		
	*n*/*N*	a*OR* (95% *CI*)	*P*-value	*n*/*N*	a*OR* (95% *CI*)	*P*-value	*n*/*N*	a*OR* (95% *CI*)	*P*-value
Sex									
Male	1574/8550	NS	NS	855/8550	1	–	2215/8550	1	–
Female	1557/8543	NS	NS	**1150/8543**	**1.51 (1.27–1.80)**	** < 0.001**	**2409/8543**	**1.16 (1.02–1.33)**	**0.02**
Age group (years)									
< 11	1151/6348	NS	NS	608/6348	1		1589/6348	1	–
11–12	959/5436	NS	NS	665/5436	1.31 (1.00–1.72)	0.05	1479/5436	1.09 (0.92–1.30)	0.31
> 12	971/5204	NS	NS	**722/5204**	**1.76 (1.40–2.22)**	** < 0.001**	**1502/5204**	**1.24 (1.00–1.53)**	**0.046**
School setting									
Rural	2508/14,249	NS	NS	1552/14,249	1	–	3655/14,249	1	–
Urban	623/2844	NS	NS	**453/844**	**1.60 (1.18–2.18)**	**0.003**	969/2844	**1.40 (1.02–1.91)**	**0.04**
Ecological zone of school									
Northern coastal	25/930	1	–	133/930	1	–	152/930	1	–
Coffee area	**1019/3303**	**23.21 (9.13–58.98)**	** < 0.001**	**228/3303**	**0.42 (0.19–0.91)**	**0.03**	**1165/3303**	**3.69 (1.72–7.89)**	**0.001**
Central highland	**2087/12,860**	**2.90 (1.20–7.02)**	**0.02**	**1644/12,860**	**0.29 (0.13–0.64)**	**0.002**	3307/12,860	0.70 (0.35–1.42)	0.32
Ethnicity									
Umbundo	1762/7620	1	–	1413/7620	1	–	2774/7620	1	–
Kikongo	**1239/7937**	**0.34 (0.24–0.49)**	** < 0.001**	**493/7937**	**0.21 (0.15–0.29)**	** < 0.001**	**1637/7937**	**0.26 (0.20–0.35)**	** < 0.001**
Kimbundo	**130/1536**	**0.40 (0.21–0.77)**	**0.006**	**99/1536**	**0.36 (0.27–0.49)**	** < 0.001**	**213/1536**	**0.35 (0.23–0.53)**	** < 0.001**
School dewormed 2013									
No	575/3061	NS	NS	355/3061	NS	NS	853/3061	NS	NS
Yes	2556/14,032	NS	NS	1650/14,032	NS	NS	3771/14,032	NS	NS
Latrines at school									
None/non-functional	1816/10,603	NS	NS	1256/10,603	NS	NS	2759/10,603	NS	NS
Functional	1315/6490	NS	NS	749/6490	NS	NS	1865/6490	NS	NS
Water at school									
Not available	2269/13,063	NS	NS	1455/13,063	NS	NS	3345/13,063	NS	NS
Available	862/4030	NS	NS	550/4030	NS	NS	1279/4030	NS	NS

*n*/*N* number of infections/at-risk population, a*OR* adjusted odds ratio, *CI* confidence interval, *NS* did not meet threshold on univariate analysis (*P* < 0.2) to be included in multivariate analysis. Based on rapid diagnostic tests (trace readings considered positive) in determining schistosomiasis, Bold statistically significant (*P *< 0.05)

Table 7 reports the factors on multivariate regression analysis associated with STH infection. For hookworm, there was a higher odds of infection for schoolchildren of Kikongo [a*OR* 68.83 (95%* CI*: 8.44–482.56), *P* < 0.01] or Kumbundo [a*OR* 50.41 (95% *CI*: 4.47–568.20), *P* = 0.002] ethnicity compared to schoolchildren of Umbundo ethnicity; while there was a lower odds of infection for schoolchildren at schools with functional latrines [a*OR* 0.35 (95% *CI*: 0.14–0.89), *P* = 0.03] compared to those at schools without functional latrines. For *A. lumbricoides*, there was a higher odds of infection for schoolchildren of Kikongo [a*OR* 6.91 (95% *CI*: 2.86–16.70), *P* < 0.001] or Kumbundo [a*OR* 9.21 (95% *CI*: 3.87–21.96), *P* < 0.001] ethnicity compared to Umbundo ethnicity, as well as for schoolchildren in the Central highland ecological zone [a*OR* 5.65 (95% *CI*: 1.79–17.82), *P* = 0.003] compared to those in the Northern coastal ecological zone. For *T. trichiura*, there was a higher odds of infection for schoolchildren of Kikongo [a*OR* 10.35 (95% *CI*: 4.25–25.22), *P* < 0.001] or Kumbundo [a*OR* 9.95 (95% *CI*: 3.30–30.01), *P* < 0.001] ethnicity compared to schoolchildren of Umbundo ethnicity, while a lower odds of infection was found for females [a*OR* 0.40 (95% *CI*: 0.23–0.69), *P* = 0.001] compared to males. Overall, the only factor associated with any STH infection was ethnicity, with a higher odds of infection for those of Kikongo [a*OR* 4.96 (95% *CI*: 2.26–10.89), *P* < 0.001] or Kumbundo [a*OR* 10.74 (95% *CI*: 4.95–23.31), *P* < 0.001] ethnicity compared to schoolchildren of Umbundo ethnicity.

**Table 7:** Factors on multivariate analysis associated with soil-transmitted helminth infection

	Hookworm			*A. lumbricoides*			*T. trichiura*			Any STH		
	*n*/*N*	a*OR* (95% *CI*)	*P*-value	*n*/*N*	a*OR* (95% *CI*)	*P*-value	*n*/*N*	a*OR* (95% *CI*)	*P*-value	*n*/*N*	a*OR* (95% *CI*)	*P*-value
**Sex**												
Male	186/1818	1	–	572/1818	NS	NS	99/1813	1	–	688/1818	1	–
Female	138/1829	0.72 (0.49–1.07)	0.11	554/1829	NS	NS	**71/1826**	**0.40 (0.23–0.69)**	**0.001**	641/1829	0.82 (0.62–1.08)	0.15
Age group (years)												
< 11	143/1394	1	–	500/1394	1	–	68/1391	1	–	576/1394	1	–
11–12	80/1096	0.74 (0.50–1.09)	0.12	315/1096	0.84 (0.63–1.12)	0.23	50/1092	1.10 (0.61–1.98)	0.31	363/1096	0.79 (0.56–1.11)	0.17
> 12	101/1090	0.82 (0.54–1.23)	0.33	310/1090	0.73 (0.44–1.21)	0.21	52/1089	0.62 (0.32–1.19)	0.15	389/1090	0.72 (0.43–1.21)	0.22
School setting												
Rural	305/3324	NS	NS	1028/3324	NS	NS	158/3316	NS	NS	1218/3324	NS	NS
Urban	19/323	NS	NS	98/323	NS	NS	12/323	NS	NS	111/323	NS	NS
Ecological zone of school												
Northern coastal	16/210	NS	NS	41/210	1	–	10/210	NS	NS	58/210	NS	NS
Coffee area	790/858	NS	NS	285/858	2.49 (0.75–8.25)	0.14	48/856	NS	NS	335/858	NS	NS
Central highland	240/2579	NS	NS	**800/2579**	**5.65 (1.79–17.82)**	**0.003**	112/2573	NS	NS	936/2579	NS	NS
Ethnicity												
Umbundo	2/1501	1	–	173/1501	1	–	15/1496	1	–	186/1501	1	–
Kikongo	**252/1818**	**63.83 (8.44–482.56)**	** < 0.001**	**754/1818**	**6.91 (2.86–16.70)**	** < 0.001**	**1672/1815**	**10.35 (4.25–25.22)**	** < 0.001**	**910/1818**	**4.96 (2.26–10.89)**	** < 0.001**
Kimbundo	**70/328**	**50.41 (4.47–568.20)**	**0.002**	**199/328**	**9.21 (3.87–21.96)**	** < 0.001**	**12/328**	**9.95 (3.30–30.01)**	** < 0.001**	**233/328**	**10.74 (4.95–23.31)**	** < 0.001**
School dewormed 2013/2014												
No	86/714	1	–	187/714	NS	NS	33/713	NS	NS	225/714	NS	NS
Yes	238/2933	0.71 (0.21–2.38)	0.57	939/2933	NS	NS	137/2926	NS	NS	1104/2933	NS	NS
Latrines at school												
None/non-functional	306/2462	1	–	874/2462	NS	NS	137/2458	NS	NS	1052/2462	NS	NS
Functional	**18/1185**	**0.35 (0.14–0.89)**	**0.03**	252/1185	NS	NS	33/1181	NS	NS	277/1185	NS	NS
Safe drinking water at school												
Not available	314/2793	1	–	987/2793	1	-	147/2789	1	–	1174/2793	1	–
Available	10/854	0.55 (0.11–2.83)	0.47	139/854	0.57 (0.22–1.46)	0.24	23/850	0.58 (0.20–1.69)	0.31	155/854	0.50 (0.19–1.30)	0.15

*n*/*N* number of infections/at-risk population, a*OR* adjusted odds ratio, *CI* confidence interval, *NS* did not meet threshold on univariate analysis (*P* < 0.2) to be included in multivariate analysis, *STH* soil-transmitted helminthining schistosomiasis, Bold statistically significant (*P* < 0.05)

## Discussion

This survey, which was carried out in 2014, represents one of the first large-scale school-based prevalence surveys using schistosomiasis RDTs, following a similar approach to the prevalence survey conducted in Namibia in 2012–2013 [12], and provides the risk-stratified rationale for the school-based preventive chemotherapy program with praziquantel and albendazole. As expected, there were differences in schistosomiasis prevalence based on whether the schistosomiasis RDT trace readings were considered positive or negative. However, this had little impact on risk stratification according to WHO-endorsed prevalence criteria, with the majority of municipalities found to be moderate to high risk schistosomiasis regions (prevalence ≥ 10%) [3] irrespective of the interpretation of trace RDT readings. As for STH infection, there was variable STH distribution across municipalities, however only two of 11 municipalities in Huambo and three of six municipalities in Zaire had a STH prevalence ≥ 20%. This may in part be due to the impact of prior deworming undertaken in schools throughout 2013. However, it is uncertain as to whether the WHO preventive chemotherapy coverage goals of at least 75% of the target population were achieved [3]. Of particular concern is the prevalence of STH infections in Uige, with 15 of 16 municipalities with a STH prevalence ≥ 20%.

How to interpret trace RDT readings in determining schistosomiasis infection across different settings has not been definitively established [22]. Of particular interest is the impact of the enhanced capacity for field work using RDTs that facilitate surveys from larger and more geographically representative populations. There was a small improvement in specificity for the RDTs when considering trace readings as negative compared to positive, as well as improved diagnostic agreement between POC-CCA® and Kato-Katz when RDT trace readings were considered negative, however there was no difference in diagnostic agreement between Hemastix® and urine filtration based on whether trace readings were considered positive or negative. Given the comparatively low detection of schistosomiasis on microscopy compared to RDTs, there is concern for the low sensitivity of microscopy methods in our survey, which hinders the ability to make more definitive conclusions as to whether RDT trace readings reflect true infection or not. The low sensitivity of microscopy methods in field surveys is well recognized, particularly in low-prevalence and light-intensity settings, which has prompted an increasing reliance on RDTs to estimate schistosomiasis prevalence [13, 16, 17, 19, 23–25]. This is in part due to the fluctuation in *S. mansoni* egg excretion and variable distribution of eggs in stool [26], with the variability in *S. mansoni* antigen excretion as detected by POC-CCA® shown to be less pronounced than egg excretion in stool for detection by Kato-Katz [27–29]. The lack of an established gold standard rapid diagnostic test for schistosomiasis and the increasingly recognized low sensitivity of Kato-Katz limits the capacity to accurately determine the prevalence of schistosomiasis and STH in the field [25, 30, 31]. Operational research is ongoing to evaluate and validate field diagnostics for schistosomiasis and STH infection, which will become increasingly important as countries strive toward elimination.

Our analysis identified both ecological zone and ethnicity to be associated with *S. mansoni* infection. Given that Umbundo ethnicity was exclusive to Huambo province in this survey, it is likely that the association with ethnicity is in part explained by other environmental, sociodemographic or behavioural differences between the provinces [32]. As such it is difficult to ascertain as to whether any cultural practices specific to schoolchildren of Umbundo ethnicity place them at higher risk for *S. mansoni* infection compared to schoolchildren of Kikongo or Kimbundo ethnicity. For *S. haematobium*, females, older age, and urban school settings were found to be risk factors in addition to ecological zone and ethnicity characteristics. Previous studies demonstrate mixed results with respect to the association of age and sex with *S. haematobium* [33–38], which likely reflects local conventions relating to the home environment that would increase exposure to water sources, such as playing, fetching water or washing [39].

The main factor identified on our analysis to be associated with STH infection was ethnicity, with schoolchildren of Kikongo or Kimbundo ethnicity more likely to be infected with any of the STH species, as well overall STH infection, compared to schoolchildren of Umbundo ethnicity. As previously discussed, the association singling out Umbundo ethnicity as a lower risk group is likely to in part reflect other environmental, sociodemographic or behavioural differences between Huambo province and the others.

There are several limitations to be acknowledged with this survey. Firstly, only a single Kato-Katz smear was conducted on stool specimens to assess for *S. mansoni* and STH species, which is not in keeping with the WHO recommendations for double Kato-Katz smears and is likely to underestimate *S. mansoni* and STH prevalence in the microscopy survey [40]. This was done to facilitate time- and cost-efficient field work given the large distances field teams had to travel throughout the provinces. This limitation is further compounded by the operator-dependent nature of microscopy, which is reliant upon training of field workers with variable experience in specimen handling and microscopy. This survey is vulnerable to selection bias associated with only selecting schoolchildren present on the day of specimen collection. This may result in selecting from a population who are well enough to attend school, which risks underestimating the prevalence of schistosomiasis and STH infection. Lastly, there was limited data collected relating to school-based water, sanitation and hygiene measures, and no data relating to home or community activities and environment for schoolchildren that could be incorporated into the risk factor analysis. However, through the inclusion of ecological zone and ethnicity variables, some broader representation of the out-of-school context for these schoolchildren is represented.

## Conclusions

This survey identified most municipalities across Huambo, Uige and Zaire met the prevalence threshold for a school-based schistosomiasis preventive chemotherapy program. As for STH, while the prevalence in many municipalities in Huambo and Zaire did not meet the WHO threshold for a school-based preventive chemotherapy program, there were a high proportion of schools that had received prior deworming. Despite this inability to discern an accurate baseline prevalence, this survey provided critical data for informing the next steps for a school-based STH preventive chemotherapy program. Ecological zone and ethnicity were factors associated with schistosomiasis and STH infection, which identifies the need for further evaluation of environmental, sociodemographic and behavioural factors at school and home to inform targeted control strategies to compliment preventive chemotherapy programs.

## Supplementary Information

**Additional file 1: Table S1.** Demographics of participants in the schistosomiasis and soil-transmitted helminth surveys across all municipalities in Huambo, Uige and Zaire provinces. **Table S2.** Prevalence of schistosomiasis based on rapid diagnostic tests and microscopy for all municipalities across Huambo, Uige and Zaire provinces. **Table S3.** Prevalence of soil-transmitted helminths for all municipalities across Huambo, Uige and Zaire provinces. **Table S4.** Comparison between trace readings on schistosomiasis rapid diagnostic tests and microscopy. **Table S5.** Results from school water, sanitation and hygiene questionnaires for all municipalities across Huambo, Uige and Zaire provinces. **Table S6.** Factors on multivariate analysis associated with schistosomiasis infection (considering RDT trace readings as negative).

## Data Availability

The datasets used and/or analysed during the current study are available from the corresponding author on reasonable request.

## Abbreviations

a*OR*: Adjusted odds ratio
*CI*: Confidence interval
DALY: Disability-adjusted life year
*IQR*: Inter-quartile range
NTD: Neglected tropical disease
POC-CCA®: Point-of-care circulating cathodic antigen
RDT: Rapid diagnostic test
STH: Soil-transmitted helminth
UNICEF: United Nations Children’s Fund
WASH: Water, sanitation and hygiene
WHO: World Health Organization

## References

[1] Bethony J, Brooker S, Albonico M, Geiger SM, Loukas A, Diemert D (2006). Soil-transmitted helminth infections: ascariasis, trichuriasis, and hookworm. Lancet.

[2] Strunz EC, Addiss DG, Stocks ME, Ogden S, Utzinger J, Freeman MC (2014). Water, sanitation, hygiene, and soil-transmitted helminth infection: a systematic review and meta-analysis. PLoS Med.

[3] World Health Organization. Helminth control in school-age children: A guide for managers of control programmes. https://apps.who.int/iris/bitstream/handle/10665/44671/9789241548267_eng.pdf?sequence=1&isAllowed=y (2011). accesssed 14 Oct 2021.

[4] Wiegand RE, Secor WE, Fleming FM, French MD, King CH, Deol AK (2021). Associations between infection intensity categories and morbidity prevalence in school-age children are much stronger for *Schistosoma haematobium* than for *S. mansoni*. PLoS Negl Trop Dis.

[5] Pullan RL, Smith JL, Jasrasaria R, Brooker SJ (2014). Global numbers of infection and disease burden of soil transmitted helminth infections in 2010. Parasit Vectors.

[6] Mawa PA, Kincaid-Smith J, Tukahebwa EM, Webster JP, Wilson S (2021). Schistosomiasis morbidity hotspots: roles of the human host, the parasite and their interface in the development of severe morbidity. Front Immunol.

[7] Hay SI, Abajobir AA, Abate KH, Abbafati C, Abbas KM, Abd-Allah F (2017). Global, regional, and national disability-adjusted life-years (DALYs) for 333 diseases and injuries and healthy life expectancy (HALE) for 195 countries and territories, 1990–2016: a systematic analysis for the Global Burden of Disease Study 2016. Lancet.

[8] World Health Organization. Ending the neglect to attain the sustainable development goals: A road map for neglected tropical diseases 2021–2030. https://www.who.int/publications/i/item/9789240010352 (2020). accessed 14 October 2021.

[9] World Health Organization. Schistosomiasis: progress report 2001–2011, strategic plan 2012 - 2020. https://apps.who.int/iris/handle/10665/78074 (2013). accessed 14 October 2021.

[10] World Health Organization. Accelerating work to overcome the global impact of neglected tropical diseases : a roadmap for implementation : executive summary. https://apps.who.int/iris/bitstream/handle/10665/70809/WHO_HTM_NTD_2012.1_eng.pdf?sequence=1&isAllowed=y (2012). accessed 14 October 2021.

[11] World Health Organization. 2030 targets for soil-transmitted helminthiases control programmes. https://apps.who.int/iris/bitstream/handle/10665/330611/9789240000315-eng.pdf?sequence=1&isAllowed=y (2020). accessed 14 October 2021. 10.1371/journal.pntd.0008505 PMC7446869 32776942

[12] Sousa-Figueiredo JC, Stanton MC, Katokele S, Arinaitwe M, Adriko M, Balfour L (2015). Mapping of schistosomiasis and soil-transmitted helminths in Namibia: the first large-scale protocol to formally include rapid diagnostic tests. PLoS Negl Trop Dis.

[13] Clark NJ, Umulisa I, Ruberanziza E, Owada K, Colley DG, Ortu G (2019). Mapping *Schistosoma mansoni* endemicity in Rwanda: a critical assessment of geographical disparities arising from circulating cathodic antigen versus Kato-Katz diagnostics. PLoS Negl Trop Dis.

[14] Knopp S, Ame SM, Hattendorf J, Ali SM, Khamis IS, Bakar F (2018). Urogenital schistosomiasis elimination in Zanzibar: accuracy of urine filtration and haematuria reagent strips for diagnosing light intensity *Schistosoma haematobium* infections. Parasit Vectors.

[15] Leta GT, Mekete K, Wuletaw Y, Gebretsadik A, Sime H, Mekasha S (2020). National mapping of soil-transmitted helminth and schistosome infections in Ethiopia. Parasit Vectors.

[16] Ortu G, Ndayishimiye O, Clements M, Kayugi D, Campbell CH, Lamine MS (2017). Countrywide reassessment of *Schistosoma mansoni* infection in Burundi using a urine-circulating cathodic antigen rapid test: informing the national control program. Am J Trop Med Hyg.

[17] Ruberanziza E, Wittmann U, Mbituyumuremyi A, Mutabazi A, Campbell CH, Colley DG (2020). Nationwide remapping of *Schistosoma mansoni* infection in Rwanda using circulating cathodic antigen rapid test: taking steps toward elimination. Am J Trop Med Hyg.

[18] Sousa-Figueiredo JC, Gamboa D, Pedro JM, Fancony C, Langa AJ, Magalhaes RJ (2012). Epidemiology of malaria, schistosomiasis, geohelminths, anemia and malnutrition in the context of a demographic surveillance system in northern Angola. PLoS ONE.

[19] Bocanegra C, Gallego S, Mendioroz J, Moreno M, Sulleiro E, Salvador F (2015). Epidemiology of schistosomiasis and usefulness of indirect diagnostic tests in school-age children in Cubal, Central Angola. PLoS Negl Trop Dis.

[20] National Section for Control of Neglected Tropical Diseases, Department of Disease Control, National Public Health Directorate, Ministry of Health, Republic of Angola. National Strategic Plan on Neglected Tropical Diseases 2017–2021. https://espen.afro.who.int/system/files/content/resources/ANGOLA_NTD_Master_Plan_2017_2021_0.pdf. accessed 6 March 2022.

[21] Sousa-Figueiredo JC, Betson M, Kabatereine NB, Stothard JR (2013). The urine circulating cathodic antigen (CCA) dipstick: a valid substitute for microscopy for mapping and point-of-care diagnosis of intestinal schistosomiasis. PLoS Negl Trop Dis.

[22] Clements MN, Donnelly CA, Fenwick A, Kabatereine NB, Knowles SCL, Meite A (2017). Interpreting ambiguous 'trace' results in *Schistosoma mansoni* CCA tests: estimating sensitivity and specificity of ambiguous results with no gold standard. PLoS Negl Trop Dis.

[23] Kittur N, Castleman JD, Campbell CH, King CH, Colley DG (2016). Comparison of *Schistosoma mansoni* prevalence and intensity of infection, as determined by the circulating cathodic antigen urine assay or by the Kato-Katz fecal assay: a systematic review. Am J Trop Med Hyg.

[24] Adriko M, Standley CJ, Tinkitina B, Tukahebwa EM, Fenwick A, Fleming FM (2014). Evaluation of circulating cathodic antigen (CCA) urine-cassette assay as a survey tool for *Schistosoma mansoni* in different transmission settings within Bugiri District, Uganda. Acta Trop.

[25] Clements MN, Corstjens P, Binder S, Campbell CH, de Dood CJ, Fenwick A (2018). Latent class analysis to evaluate performance of point-of-care CCA for low-intensity *Schistosoma mansoni* infections in Burundi. Parasit Vectors.

[26] Krauth SJ, Coulibaly JT, Knopp S, Traore M, N'Goran EK, Utzinger J (2012). An in-depth analysis of a piece of shit: distribution of *Schistosoma mansoni* and hookworm eggs in human stool. PLoS Negl Trop Dis.

[27] Van Etten L, Engels D, Krijger FW, Nkulikyinka L, Gryseels B, Deelder AM (1996). Fluctuation of schistosome circulating antigen levels in urine of individuals with *Schistosoma mansoni* infection in Burundi. Am J Trop Med Hyg.

[28] Polman K, Engels D, Fathers L, Deelder AM, Gryseels B (1998). Day-to-day fluctuation of schistosome circulating antigen levels in serum and urine of humans infected with *Schistosoma mansoni* in Burundi. Am J Trop Med Hyg.

[29] Mwinzi PN, Kittur N, Ochola E, Cooper PJ, Campbell CH, King CH (2015). Additional evaluation of the point-of-contact circulating cathodic antigen assay for *Schistosoma mansoni* infection. Front Public Health.

[30] Amoah AS, Hoekstra PT, Casacuberta-Partal M, Coffeng LE, Corstjens P, Greco B (2020). Sensitive diagnostic tools and targeted drug administration strategies are needed to eliminate schistosomiasis. Lancet Infect Dis.

[31] Cavalcanti MG, Cunha AFA, Peralta JM (2019). The advances in molecular and new point-of-care (POC) diagnosis of schistosomiasis pre- and post-praziquantel use: in the pursuit of more reliable approaches for low endemic and non-endemic areas. Front Immunol.

[32] Pinot de Moira A, Fulford AJ, Kabatereine NB, Ouma JH, Booth M, Dunne DW (2010). Analysis of complex patterns of human exposure and immunity to schistosomiasis mansoni: the influence of age, sex, ethnicity and IgE. PLoS Negl Trop Dis.

[33] Augusto G, Nala R, Casmo V, Sabonete A, Mapaco L, Monteiro J (2009). Geographic distribution and prevalence of schistosomiasis and soil-transmitted helminths among schoolchildren in Mozambique. Am J Trop Med Hyg.

[34] Kabuyaya M, Chimbari MJ, Manyangadze T, Mukaratirwa S (2017). Efficacy of praziquantel on *Schistosoma haematobium* and re-infection rates among school-going children in the Ndumo area of uMkhanyakude district, KwaZulu-Natal, South Africa. Infect Dis Poverty.

[35] Geleta S, Alemu A, Getie S, Mekonnen Z, Erko B (2015). Prevalence of urinary schistosomiasis and associated risk factors among Abobo Primary School children in Gambella Regional State, southwestern Ethiopia: a cross sectional study. Parasit Vectors.

[36] Kapito-Tembo AP, Mwapasa V, Meshnick SR, Samanyika Y, Banda D, Bowie C (2009). Prevalence distribution and risk factors for *Schistosoma hematobium* infection among school children in Blantyre, Malawi. PLoS Negl Trop Dis.

[37] Degarege A, Mekonnen Z, Levecke B, Legesse M, Negash Y, Vercruysse J (2015). Prevalence of *Schistosoma haematobium* infection among school-age children in Afar Area, Northeastern Ethiopia. PLoS ONE.

[38] Ismail HA, Hong ST, Babiker AT, Hassan RM, Sulaiman MA, Jeong HG (2014). Prevalence, risk factors, and clinical manifestations of schistosomiasis among school children in the White Nile River basin. Sudan Parasit Vectors.

[39] Kabuyaya M, Chimbari MJ, Manyangadze T, Mukaratirwa S (2017). Schistosomiasis risk factors based on the infection status among school-going children in the Ndumo area, uMkhanyakude district, South Africa. South Afr J Infect Dis.

[40] Levecke B, Brooker SJ, Knopp S, Steinmann P, Sousa-Figueiredo JC, Stothard JR (2014). Effect of sampling and diagnostic effort on the assessment of schistosomiasis and soil-transmitted helminthiasis and drug efficacy: a meta-analysis of six drug efficacy trials and one epidemiological survey. Parasitology.

