# Supplementary material for: Burden and factors associated with schistosomiasis and soil-transmitted helminth infections among school-age children in Huambo, Uige and Zaire provinces, Angola
Source: Infect Dis Poverty. 2022 Jun 25;11:73. doi: 10.1186/s40249-022-00975-z (PMC9233808; doi:10.1186/s40249-022-00975-z)
Supplement: Supplementary file 1 — Additional file 1: Table S1. Demographics of participants in the schistosomiasis and soil-transmitted helminth surveys across all municipalities in Huambo, Uige and Zaire provinces. Table S2. Prevalence of schistosomiasis based on rapid diagnostic tests and microscopy for all municipalities across Huambo, Uige and Zaire provinces. Table S3. Prevalence of soil-transmitted helminths for all municipalities across Huambo, Uige and Zaire provinces. Table S4. Comparison between trace readings on schistosomiasis rapid diagnostic tests and microscopy. Table S5. Results from school water, sanitation and hygiene questionnaires for all municipalities across Huambo, Uige and Zaire provinces. Table S6. Factors on multivariate analysis associated with schistosomiasis infection (considering RDT trace readings as negative). [file 40249_2022_975_MOESM1_ESM.docx]

**Additional File 1.** Demographics of participants in the schistosomiasis and soil-transmitted helminth surveys across all municipalities in Huambo, Uige and Zaire provinces.

|  | **Rapid diagnostic testing survey** | | | | | **Microscopy survey** | | | | |
| --- | --- | --- | --- | --- | --- | --- | --- | --- | --- | --- |
|  | **Schools**  ***N*** | **Students** | | | | **Schools**  ***N*** | **Students** | | | |
|  |  | **Total**  ***N*** | **Male**  ***N* (%)** | **Female**  ***N* (%)** | **Age, years**  **Median (*IQR*)** |  | **Total**  ***N*** | **Male**  ***N* (%)** | **Female**  ***N* (%)** | **Age, years**  **Median (*IQR*)** |
| **Huambo province** | **254** | **7,602** | **3,796 (49.2)** | **3,824 (50.2)** | **11 (10–13)** | **50** | **1,501** | **750 (50)** | **751 (50)** | **11 (10–13)** |
| Bailundo | 24 | 720 | 361 (50.1) | 359 (49.9) | 12 (10–13) | 5 | 150 | 75 (50) | 75 (50) | 12.5 (11–14) |
| Caala | 38 | 1,140 | 570 (50) | 570 (50) | 11 (10–13) | 7 | 211 | 105 (49.8) | 106 (50.2) | 11 (10–12) |
| Cachiungo | 27 | 810 | 405 (50) | 405 (50) | 11 (10–13) | 5 | 150 | 75 (50) | 75 (50) | 12 (10–14) |
| Ekunha | 13 | 390 | 195 (50) | 195 (50) | 11 (10–13) | 3 | 90 | 45 (50) | 45 (50) | 11 (10–13) |
| Huambo | 52 | 1,560 | 765 (49) | 795 (51) | 12 (10–13) | 11 | 330 | 165 (50) | 165 (50) | 12 (11–13) |
| Londuimbale | 20 | 600 | 300 (50) | 300 (50) | 11 (10–13) | 4 | 120 | 60 (50) | 60 (50) | 11 (10–13) |
| Logonjo | 24 | 720 | 360 (50) | 360 (50) | 11 (10–13) | 5 | 150 | 75 (50) | 75 (50) | 10 (10–12) |
| Mungo | 5 | 150 | 75 (50) | 75 (50) | 11 (10–13) | 1 | 30 | 15 (50) | 15 (50) | 11 (10–12) |
| Tchikala-Tcholohanga | 21 | 630 | 315 (50) | 315 (50) | 11 (10–12) | 4 | 120 | 60 (50) | 60 (50) | 11 (10–12) |
| Tchinjenje | 16 | 480 | 240 (50) | 240 (50) | 11 (10–13) | 2 | 60 | 30 (50) | 30 (50) | 12 (10–12) |
| Ukuma | 14 | 420 | 210 (50) | 210 (50) | 11 (10–13) | 3 | 90 | 45 (50) | 45 (50) | 10 (10–11) |
| **Uige province** | **265** | **7,793** | **3,916 (50.3)** | **3,877 (49.8)** | **11 (10–13)** | **60** | **1,818** | **903 (49.7)** | **915 (50.3)** | **11 (9–13)** |
| Ambuila | 9 | 256 | 135 (52.7) | 121 (47.3) | 11 (10–13) | 2 | 60 | 30 (50) | 30(50) | 12 (10–13) |
| Bembe | 14 | 450 | 225 (50) | 225 (50) | 11 (10–13) | 3 | 90 | 45 (50) | 45 (50) | 12 (9–13) |
| Buengas | 22 | 644 | 330 (51.2) | 314 (48.8) | 11 (10–13) | 4 | 120 | 60 (50) | 60 (50) | 12 (10–13) |
| Bungo | 14 | 413 | 210 (50.9) | 203 (49.2) | 11 (10–13) | 3 | 90 | 45 (50) | 45 (50) | 11 (10–12) |
| Cangola | 18 | 540 | 270 (50) | 270 (50) | 10 (10–12) | 4 | 120 | 60 (50) | 60 (50) | 10 (9–12) |
| Damba | 22 | 659 | 330 (50.1) | 329 (49.9) | 11 (10–13) | 4 | 120 | 60 (50) | 60 (50) | 10 (8–12) |
| Maquela do Zombo | 21 | 629 | 315 (50.1) | 314 (49.9) | 12 (10–14) | 5 | 150 | 75 (50) | 75 (50) | 11 (9–13) |
| Milunga | 10 | 283 | 133 (47) | 150 (53) | 12 (10–14) | 2 | 60 | 30 (50) | 30 (50) | 14 (13–15) |
| Mucaba | 13 | 353 | 195 (55.2) | 158 (44.8) | 11 (10–12) | 3 | 90 | 45 (50) | 45 (50) | 10 (8–11) |
| Negage | 18 | 516 | 263 (51) | 253 (49) | 12 (11–14) | 3 | 90 | 45 (50) | 45 (50) | 11.5 (10–13) |
| Puri | 10 | 300 | 135 (45) | 165 (55) | 10 (8–11) | 2 | 60 | 30 (50) | 30 (50) | 8 (6–10) |
| Quimbele | 24 | 673 | 345 (51.3) | 328 (48.7) | 11 (10–13) | 5 | 150 | 75 (50) | 75 (50) | 10 (7–11) |
| Quitexe | 6 | 180 | 90 (50) | 90 (50) | 10 (8–11) | 2 | 60 | 30 (50) | 30 (50) | 10 (9–12) |
| Sanza Pombo | 30 | 889 | 442 (49.7) | 447 (50.3) | 11 (10–13) | 5 | 150 | 75 (50) | 75 (50) | 12 (10–14) |
| Songo | 14 | 420 | 210 (50) | 210 (50) | 10 (9–12) | 2 | 90 | 45 (50) | 45 (50) | 11 (8–12) |
| Uige | 20 | 588 | 288 (49) | 300 (51) | 11 (10–13) | 11 | 318 | 153 (48.1) | 165 (51.9) | 11 (10–13) |
| **Zaire province** | **56** | **1,680** | **838 (49.9)** | **842 (50.1)** | **12 (10–13)** | **11** | **330** | **165 (50)** | **165 (50)** | **12 (10–14)** |
| Cuimba | 12 | 360 | 180 (50) | 180 (50) | 13 (11–14) | 2 | 60 | 30 (50) | 30 (50) | 13 (12–14) |
| Mbanza Congo | 13 | 390 | 195 (50) | 195 (50) | 12 (10–14) | 2 | 60 | 30 (50) | 30 (50) | 12 (10–14) |
| Noqui | 9 | 270 | 136 (50.4) | 134 (49.6) | 13 (11–14) | 2 | 60 | 30 (50) | 30 (50) | 13 (11–14) |
| Nzeto | 5 | 150 | 75 (50) | 75 (50) | 11 (10–13) | 1 | 30 | 15 (50) | 15 (50) | 10 (8–12) |
| Soyo | 11 | 330 | 162 (49.1) | 168 (50.9) | 12 (10–13) | 3 | 90 | 45 (50) | 45 (50) | 12 (11–13) |
| Tomboco | 6 | 180 | 90 (50) | 90 (50) | 11.5 (10–13) | 1 | 30 | 15 (50) | 15 (50) | 12 (12–13) |
| **Total** | **575** | **17,093** | **8,550 (50.0)** | **8,543 (50.0)** | **11 (10–13)** | **121** | **3,649** | **1,818 (49.8)** | **1,831 (50.2)** | **11 (10–13)** |

*N*: number of schools / schoolchildren participating in the survey. *IQR*: interquartile range. yrs: years.

**Additional File 2.** Prevalence of schistosomiasis based on rapid diagnostic tests and microscopy for all municipalities across Huambo, Uige and Zaire provinces.

|  | **Rapid diagnostic tests** | | | | | | | **Microscopy** | | | | |
| --- | --- | --- | --- | --- | --- | --- | --- | --- | --- | --- | --- | --- |
|  | ***N*** | ***S. mansoni***  **% (95% *CI*)** | | ***S. haematobium***  **% (95% *CI*)** | | **Any schistosomiasis**  **% (95% *CI*)** | | ***S. mansoni*** | | ***S. haematobium*** | | **Any schistosomiasis**  **% (95% *CI*)** |
|  |  | **Trace = pos** | **Trace = neg** | **Trace = pos** | **Trace = neg** | **Trace = pos** | **Trace = neg** | ***N*** | **%**  **(95% *CI*)** | ***N*** | **%**  **(95% *CI*)** |  |
| **Huambo** | **7,620** | **20.8**  **(17.4–24.6))** | **8.9**  **(7.3–10.7)** | **18.7**  **(15.9–21.8)** | **11.9**  **(9.9–14.3)** | **34.7**  **(30.7–38.8)** | **19.1**  **(16.7–21.9)** | **1,501** | **0.2**  **(0.05–0.9)** | **1,500** | **8.4**  **(4.9–14.0)** | **8.6**  **(0.5–14.2)** |
| Bailundo | 720 | 25.2  (15.8–37.6) | 10.8  (6.7–16.9) | 12.1  (8.0–18.0) | 7.1  (4.1–12.0) | 32.8  (22.5–45.1) | 15.9  (11.0–22.3) | 150 | 0 | 150 | 3.3  (0.8–12.3) | 3.3  (0.8–12.3) |
| Caala | 1,140 | 10.9  (5.0–22.2) | 5.2  (2.1–12.3) | 29.8  (19.8–42.2) | 20.8  (13.3–31.1) | 36.1  (24.4–49.8) | 25.1  (16.2–36.8) | 211 | 0 | 210 | 19.6  (6.5–46.0) | 19.6  (6.5–46.0) |
| Cachiungo | 810 | 31.2  (20.4–44.5) | 12.9  (7.6–20.9) | 13.2  (9.4–18.2) | 7.4  (5.0–10.9) | 39.0  (28.8–50.3) | 17.6  (11.8–25.4) | 150 | 0 | 150 | 5.4  (3.3–8.6) | 5.4  (3.3–8.6) |
| Ekunha | 390 | 26.8  (11.9–50.0) | 11.6  (4.7–25.7) | 8.5  (2.4–26.1) | 5.5  (1.4–19.0) | 31.1  (12.9–58.0) | 15.6  (6.0–35.0) | 90 | 0 | 90 | 0 | 0 |
| Huambo | 1,560 | 6.6  (4.5–9.6) | 2.7  (1.5–4.8) | 22.7  (17.3–29.1) | 14.5  (10.6–19.4) | 26.9  (20.9–34.0) | 16.1  (12.2–21.0) | 330 | 0.8  (0.2–3.5) | 330 | 8.9  (2.5–27.1) | 9.7  (3.0–27.1) |
| Londuimbali | 600 | 38.9  (27.7–51.5) | 15.8  (11.2–21.8) | 15.6  (9.3–25.0) | 6.3  (3.1–12.4) | 47.2  (35.6–59.0) | 20.7  (15.2–27.5) | 120 | 0 | 120 | 2.6  (0.4–1.5) | 2.6  (0.4–1.5) |
| Logonjo | 720 | 26.4  (14.5–43.3) | 12.9  (7.3–21.8) | 15.6  (8.3–27.3) | 10.1  (5.2–18.6) | 35.6  (20.4–54.4) | 21.5  (12.4–34.6) | 150 | 0 | 150 | 7.6  (0.3–71.1) | 7.6  (0.3–71.1) |
| Mungo | 150 | 31.1  (8.3–69.1) | 12.5  (4.4–30.6) | 0 | 0 | 31.1  (8.3–69.1) | 12.5  (4.4–30.6) | 30 | 0 | 30 | 0 | 0 |
| Tchikala-Tcholohanga | 630 | 35.4  (22.3–51.1) | 14.2  (8.1–23.7) | 17.5  (8.2–33.5) | 12.6  (5.3–27.0) | 46.4  (32.3–61.1) | 24.2  (14.7–37.3) | 120 | 0 | 120 | 34.4  (11.5–95.9) | 34.4  (1.2–95.9) |
| Tchinjenje | 480 | 20.9  (10.0–38.6) | 11.3  (6.0–20.3) | 25.9  (14.9–41.3) | 20.4  (10.5–35.8) | 39.4  (23.7–57.8) | 28.2  (16.1–44.5) | 60 | 0 | 60 | 6.7  (6.7–6.7) | 6.7  (6.7–6.7) |
| Ukuma | 420 | 47.2  (32.4–62.5) | 18.4  (11.9–27.4) | 26.5  (15.6–41.3) | 19.2  (10.9–31.7) | 57.0  (38.5–73.8) | 31.5  (21.9–42.9) | 90 | 0 | 90 | 22.2  (2.7–74.3) | 22.2  (2.7–74.3) |
| **Uige** | **7,793** | **21.2**  **(15.6–28.2)** | **16.1**  **(11.6–21.8)** | **5.6**  **(4.6–6.7)** | **2.8**  **(2.2–3.5)** | **25.3**  **(19.8–31.9)** | **18.4**  **(13.8–24.1)** | **1,818** | **20.0**  **(9.1–38.6)** | **1,615** | **0.7**  **(0.3–1.8)** | **24.7**  **(12.6–42.8)** |
| Ambuila | 256 | 23.0  (9.0–47.3) | 14.9  (5.7–33.4) | 5.3  (1.9–14.5) | 2.2  (0.9–5.2) | 26.8  (13.0–47.4) | 17.0  (8.1–32.3) | 60 | 14.3  (0.4–87.8) | 60 | 0 | 14.3  (0.4–87.8) |
| Bembe | 450 | 24.9  (18.6–32.5) | 18.6  (13.2–25.7) | 9.3  (3.1–24.9) | 7.4  (2.3–21.4) | 31.5  (21.7–43.4) | 23.9  (17.6–31.5) | 90 | 25.4  (0.2–98.0) | 90 | 5.9  (1.4–22.4) | 29.6  (0.4–97.5) |
| Buengas | 644 | 3.4  (1.4–7.9) | 2.3  (0.7–6.8) | 2.5  (1.2–5.1) | 1.4  (0.5–3.6) | 5.9  (2.9–11.5) | 3.6  (1.3–9.4) | 120 | 25.0  (0.1–40.7) | 120 | 1.7  (0.1–30.6) | 4.2  (0.2–55.2) |
| Bungo | 413 | 9.1  (5.6–14.6) | 7.6  (5.1–11.2) | 3.6  (0.9–12.6) | 2.2  (0.5–9.2) | 12.4  (6.3–23.0) | 9.6  (5.5–16.2) | 90 | 7.8  (3.1–18.2) | 90 | 0 | 7.8  (3.1–18.2) |
| Cangola | 540 | 3.8  (1.6–8.7) | 2.9  (1.2–7.0) | 5.7  (3.0–10.6) | 9.6  (0.3–2.9) | 9.2  (7.0–11.9) | 3.9  (1.6–8.8) | 120 | 1.7  (0.04–40.4) | 120 | 0.7  (0.02–18.7) | 2.4  (0.2–26.8) |
| Damba | 659 | 7.0  (5.1–9.5) | 5.1  (3.4–7.8) | 3.9  (2.6–5.8) | 2.6  (1.5–4.6) | 10.4  (7.9–13.6) | 7.3  (4.7–11.0) | 120 | 3.3  (1.4–7.4) | 120 | 0 | 3.3  (1.4–7.4) |
| Maquela do Zombo | 629 | 9.9  (7.1–13.6) | 7.9  (5.2–11.8) | 6.6  (3.5–12.2) | 3.4  (1.5–7.8) | 15.2  (10.3–22.0) | 10.3  (6.4–16.1) | 150 | 2.3  (0.9–6.1) | 150 | 0 | 2.3  (0.9–6.1) |
| Milunga | 283 | 4.4  (1.7–10.6) | 3.6  (1.3–9.4) | 4.5  (2.2–9.2) | 3.2  (1.6–6.3) | 8.9  (4.2–17.9) | 6.8  (3.0–14.7) | 60 | 2.4  (0–98.1) | 57 | 0 | 2.4  (0–98.1) |
| Mucaba | 353 | 14.6  (5.7–32.8) | 11.9  (3.7–32.1) | 0.7  (0.1–4.3) | 0.3  (0.05–1.6) | 15.2  (6.1–32.9) | 12.0  (3.8–32.1) | 90 | 4.4  (1.0–17.4) | 90 | 0 | 4.4  (1.0–17.4) |
| Negage | 516 | 14.9  (8.9–23.9) | 11.8  (6.5–20.5) | 9.9  (7.4–13.2) | 3.8  (2.5–5.6) | 23.1  (16.2–31.9) | 14.8  (8.9–23.4) | 90 | 10.9  (3.3–30.2) | 88 | 2.7  (0.1–10.7) | 13.6  (3.9–37.6) |
| Puri | 300 | 6.1  (2.7–13.0) | 4.1  (1.4–11.5) | 3.7  (0.8–15.7) | 1.6  (0.4–6.3) | 9.3  (3.9–20.8) | 5.7  (2.2–13.7) | 60 | 0 | 60 | 0 | 0 |
| Quimbele | 673 | 2.0  (0.8–4.9) | 0.9  (0.3–2.2) | 4.2  (2.6–6.7) | 2.7  (1.7–4.1) | 6.1  (4.0–9,4) | 3.5  (2.4–5.3) | 150 | 0 | 150 | 1.2  (0.1–18.7) | 1.2  (0.1–18.7) |
| Quitexe | 180 | 13.5  (5.1–31.4) | 4.9  (2.2–10.3) | 8.4  (3.9–17.0) | 4.2  (1.7–10.4) | 18.7  (7.7–38.9) | 7.8  (3.6–15.9) | 60 | 0 | 60 | 0 | 0 |
| Sanza Pombo | 889 | 6.6  (4.9–8.8) | 4.3  (3.1–5.9) | 7.0  (4.8–10.1) | 4.0  (2.5–6.6) | 12.9  (9.7–16.9) | 8.3  (6.1–11.1) | 150 | 2.7  (0.5–12.7) | 150 | 1.9 (0.2–20.1) | 4.7  (0.6–26.9) |
| Songo | 420 | 74.8  (56.3–87.3) | 55.0  (38.5–70.4) | 7.6  (3.8–14.8) | 1.6  (0.5–5.4) | 77.3  (58.8–89.0) | 55.6  (39.7–70.4) | 90 | 87.4  (7.7–99.8) | 90 | 0 | 87.4  (7.7–99.8) |
| Uige | 588 | 58.7  (40.6–74.7) | 45.4  (33.8–57.6) | 4.5  (2.0–9.9) | 2.4  (1.1–5.0) | 60.1  (42.6–75.3) | 47.7  (35.7–60.0) | 318 | 34.1  (12.6–65.0) | 120 | 0 | 34.1  (12.6–65.0) |
| **Zaire** | **1,680** | **23.1**  **(11.8–40.5)** | **14.1**  **(6.9–26.6)** | **11.3**  **(7.6–16.7)** | **7.0**  **(4.6–10.5)** | **32.2**  **(20.5–46.7)** | **20.1**  **(12.4–31.1)** | **330** | **0.1**  **(0.09–1.1)** | **330** | **3.3**  **(5.3–17.9)** | **3.3**  **(0.5–17.9)** |
| Cuimba | 360 | 21.8  (15.6–29.6) | 11.7  (7.5–17.8) | 10.4  (4.3–23.1) | 6.4  (2.2–17.1) | 29.2  (21.5–38.2) | 15.8  (9.6–24.9) | 60 | 0 | 60 | 0 | 0 |
| Mbanza Congo | 390 | 47.1  (24.8–70.6) | 29.9  (16.1–48.7) | 7.4  (5.1–10.7) | 4.9  (2.9–8.2) | 51.2  (28.2–73.7) | 34.1  (19.7–52.0) | 60 | 1.3  (0–100) | 60 | 2.7  (0–100) | 2.7  (0–100) |
| Noqui | 270 | 2.8  (0.7–10.8) | 1.8  (0.5–5.9) | 31.2  (14.2–55.5) | 18.0  (10.3–29.8) | 32.2  (14.9–56.4) | 19.4  (11.0–32.0) | 60 | 0 | 60 | 0 | 0 |
| Nzeto | 150 | 12.8  (1.6–56.9) | 9.7  (1.3–46.3) | 17.0  (3.6–53.0) | 15.2  (2.1–59.7) | 26.8  (4.1–75.9) | 24.9  (2.9–78.9) | 30 | 0 | 30 | 3.3  (0–100) | 3.3  (0–100) |
| Soyo | 330 | 0.9  (0.1–5.7) | 0 | 6.0  (2.4–14.2) | 4.1  (1.8–8.9) | 6.9  (3.2–14.4) | 4.1  (1.8–8.9) | 90 | 0 | 90 | 6.6  (0.1–83.0) | 6.6  (0.1–83.0) |
| Tomboco | 180 | 3.7  (5.4–21.4) | 1.6  (0.2–14.2) | 3.8  (0.9–14.2) | 0 | 7.5  (3.0–17.7) | 1.6  (0.2–14.2) | 30 | 0 | 30 | 0 | 0 |
| **Total** | **17,093** | **21.2**  **(18.1–24.6)** | **11.8**  **(9.7–14.3)** | **13.6**  **(11.9–15.6)** | **8.4**  **(7.1–9.9)** | **31.4**  **(28.2–34.7)** | **19.0**  **(16.7–21.5)** | **3,649** | **8.9**  **(3.6–20.5)** | **3,445** | **5.0**  **(3.1–8.0)** | **14.5**  **(8.2–24.4)** |

*N*: number of schoolchildren providing specimens for the respective surveys. *CI*: confidence interval. Adjusted for clustering at school level.

**Additional File 3.** Prevalence of soil-transmitted helminths for all municipalities across Huambo, Uige and Zaire provinces.

|  | ***N*** | **Hookworm**  **% (95% *CI*)** | ***A. lumbricoides***  **% (95% *CI*)** | ***T. trichiura***  **% (95% *CI*)** | **Any STH**  **% (95% *CI*)** |
| --- | --- | --- | --- | --- | --- |
| **Huambo province** | **1,501** | **0.1 (0.02–1.1)** | **12.5 (6.7–22.0)** | **0.7 (0.3–1.5)** | **13.1 (7.2–22.7)** |
| Bailundo | 150 | 0 | 2.6 (1.0–6.4) | 0.2 (0.01–4.9) | 2.8 (1.2–6.4) |
| Caala | 211 | 0 | 13.1 (7.3–22.6) | 0 | 13.1 (7.3–22.6) |
| Cachiungo | 150 | 0 | 4.0 (2.4–6.5) | 0 | 4.0 (2.4–6.5) |
| Ekunha | 90 | 0 | 17.6 (0.3–93.4) | 4.2 (0.3–38.0) | 21.8 (0.4–94.6) |
| Huambo | 330 | 0.6 (0.1–5.2) | 32.8 (17.1–53.4) | 0.7 (0.2–3.1) | 33.2 (17.7–53.7) |
| Londuimbale | 120 | 0 | 0.8 (0.03–17.3) | 0 | 0.8 (0.03–17.3) |
| Logonjo | 150 | 0 | 4.0 (0.2–48.0) | 0 | 3.7 (0.2–48.0) |
| Mungo | 30 | 0 | 0 | 3.3 (0–100) | 3.3 (1–100) |
| Tchikala-Tcholohanga | 120 | 0 | 3.7 (0.3–30.9) | 0 | 3.7 (0.3–30.9) |
| Tchinjenje | 60 | 0 | 0 | 3.8 (0–100) | 3.8 (0–100) |
| Ukuma | 90 | 0 | 6.0 (0.7–37.1) | 1.8 (0.01–69.4) | 7.7 (0.4–61.0) |
| **Uige province** | **1,818** | **11.1 (7.2–16.8)** | **40.8 (31.1–51.3)** | **7.1 (5.2–9.7)** | **49.4 (38.7–60.1)** |
| Ambuila | 60 | 4.9 (0–97.4) | 7.6 (0.01–98.3) | 18.9 (0.02–99.6) | 30.6 (1.1–94.3) |
| Bembe | 90 | 0.9 (0.01–52.6) | 3.4 (0.02–84.4) | 0.9 (0.01–52.6) | 5.2 (0.2–63.0) |
| Buengas | 120 | 11.8 (2.3–42.7) | 71.8 (19.3–96.4) | 24.2 (8.3–52.9) | 75.6 (17.8–978) |
| Bungo | 90 | 10.7 (2.2–39.2) | 62.1 (12.4–95.0) | 12.8 (1.8–54.1) | 76.1 (25.7–96.7) |
| Cangola | 120 | 42.6 (14.0–77.2) | 29.9 (2.5–87.8) | 0.5 (0.0–17.4) | 56.2 (16.5–89.3) |
| Damba | 120 | 37.3 (6.4–83.7) | 62.0 (35.1–83.1) | 5.3 (1.3–18.9) | 73.3 (44.0–90.6) |
| Maquela do Zombo | 150 | 20.6 (15.5–26.9) | 38.2 (4.5–89.0) | 14.1 (3.9–40.0) | 54.3 (16.2–88.0) |
| Milunga | 60 | 17.6 (6.0–41.8) | 30.6 (0–100) | 0 | 43.3 (0–100) |
| Mucaba | 90 | 13.3 (4.9–82.7) | 65.8 (39.9–84.7) | 3.7 (0.2–46.4) | 70.1 (40.3–87.5) |
| Negage | 90 | 0 | 61.0 (30.8–84.6) | 10.8 (8.5–13.5) | 61.0 (30.8–84.6) |
| Puri | 60 | 4.0 (0–100) | 58.0 (1.6–99.1) | 0 | 61.3 (22.4–89.7) |
| Quimbele | 150 | 23.3 (14.2–35.9) | 81.4 (74.3–86.9) | 7.4 (2.7–19.0) | 84.0 (74.2–90.6) |
| Quitexe | 60 | 6.1 (0–100) | 89.7(24.2–100) | 10.2 (0–100) | 89.7 (24.2–100) |
| Sanza Pombo | 150 | 41.4 (21.6–64.5) | 14.8 (5.6–33.8) | 0 | 47.1 (28.1–67.0) |
| Songo | 90 | 9.0 (0.05–95.3) | 65.3 (13.7–95.7) | 4.0 (0.03–87.3) | 72.3 (35.6–92.5) |
| Uige | 318 | 4.1 (1.1–13.7) | 29.4 (12.5–54.7) | 5.5 (2.6–11.0) | 34.7 (14.6–62.2) |
| **Zaire province** | **330** | **4.2 (0.9–18.1)** | **16.5 (8.7–28.9)** | **2.4 (0.8–6.7)** | **20.6 (10.6–36.2)** |
| Cuimba | 60 | 0 | 21.3 (3.0–70.3) | 0 | 21.3 (3.0–70.3) |
| Mbanza Congo | 60 | 0 | 10.2 (0–100) | 2.6 (0–100) | 12.8 (0.01–97.0) |
| Noqui | 60 | 8.0 (0–100) | 7.4 (0–100) | 2.5 (0–93.4) | 13.8 (0–100) |
| Nzeto | 30 | 0 | 0 | 6.7 (0–100) | 6.7 (0–100) |
| Soyo | 90 | 5.2 (0.5–39.0) | 30.9 (35.3–84.6) | 7.4 (0.4–59.4) | 36.8 (4.0–89.1) |
| Tomboco | 30 | 6.7 (0–100) | 13.3 (0–100) | 0 | 20 (0–100) |
| **Total** | **3,647** | **5.2 (3.7–7.3)** | **25.1 (19.4–31.9)** | **3.6 (2.6–5.0)** | **29.5 (23.2–36.6)** |

*N*: number of schoolchildren providing specimens. *CI*: confidence interval. STH: soil-transmitted helminth. Adjusted for clustering at school level.

**Additional File 4.** Comparison between trace readings on schistosomiasis rapid diagnostic tests and microscopy.

|  | **POC-CCA^®^ trace reading**  ***N* = 225** | **Hemastix^®^ trace reading**  ***N* = 96** |
| --- | --- | --- |
|  | ***n* (%)** | ***n* (%)** |
| **Kato-Katz** |  |  |
| Negative | 220 (97.8) | - |
| Light intensity | 5 (2.2) | - |
| Moderate intensity | 0 (0) | - |
| Heavy intensity | 0 (0) | - |
| **Urine filtration** |  |  |
| Negative | - | 72 (75.0) |
| Light intensity | - | 18 (18.8) |
| Heavy intensity | - | 6 (6.2) |

POC-CCA^®^ = point of care circulating cathodic antigen.

**Additional File 5.** Results from school water, sanitation and hygiene questionnaires for all municipalities across Huambo, Uige and Zaire provinces.

|  | **Schools** | **Bathrooms** | | **Safe drinking water source** | | | **Dewormed in 2013**  ***n* (%)** |
| --- | --- | --- | --- | --- | --- | --- | --- |
|  | ***N*** | **Available**  ***n* (%)** | **In good condition**  ***n* (%)** | **Reliable**  ***n* (%)** | **Tap**  ***n* (%)** | **Hole/other**  ***n* (%)** |  |
| **Huambo province** | **254** | **148 (58.3)** | **124 (48.8)** | **86 (33.9)** | **63 (24.8)** | **23 (9.1)** | **203 (79.9)** |
| Bailundo | 24 | 17 (70.8) | 13 (54.2) | 7 (29.2) | 5 (20.8) | 2 (8.3) | 22 (91.7) |
| Caala | 38 | 14 (36.8) | 12 (31.6) | 12 (31.6) | 10 (26.3) | 2 (5.3) | 30 (79.0) |
| Cachiungo | 27 | 20 (74.1) | 16 (59.3) | 11 (40.7) | 8 (29.6) | 3 (11.1) | 19 (70.4) |
| Ekunha | 13 | 8 (61.5) | 8 (61.5) | 3 (23.1) | 1 (7.7) | 2 (15.4) | 10 (76.9) |
| Huambo | 52 | 40 (76.9) | 32 (61.5) | 23 (44.2) | 19 (36.5) | 4 (7.7) | 47 (90.4) |
| Londuimbale | 20 | 10 (50.0) | 10 (50.0) | 3 (15.0) | 3 (15.0) | 0 | 17 (85.0) |
| Logonjo | 24 | 10 (41.7) | 10 (41.7) | 7 (29.2) | 5 (20.8) | 2 (8.3) | 19 (79.2) |
| Mungo | 5 | 4 (80.0) | 4 (80.) | 4 (80.0) | 3 (60.0) | 1 (20.0) | 5 (100) |
| Tchikala-Tcholohanga | 21 | 9 (42.9) | 8 (38.1) | 8 (38.1) | 4 (19.1) | 4 (19.1) | 11 (52.4) |
| Tchinjenje | 16 | 6 (37.5) | 3 (18.8) | 5 (31.3) | 3 (18.8) | 2 (12.5) | 11 (68.8) |
| Ukuma | 14 | 10 (71.4) | 8 (57.1) | 3 (21.4) | 2 (14.3) | 1 (7.1) | 12 (85.7) |
| **Uige province** | **265** | **127 (47.4)** | **81 (30.5)** | **40 (15.0)** | **20 (7.5)** | **20 (7.5)** | **218 (82.3)** |
| Ambuila | 9 | 5 (55.6) | 3 (33.3) | 1 (11.1) | 0 | 1 (11.1) | 7 (77.8) |
| Bembe | 14 | 7 (46.7) | 6 (40.0) | 3 (20.0) | 1 (6.7) | 2 (13.3) | 13 (86.7) |
| Buengas | 22 | 9 (40.9) | 2 (9.1) | 1 (4.6) | 0 | 1 (4.6) | 20 (90.9) |
| Bungo | 14 | 7 (50.0) | 4 (28.6) | 1 (7.1) | 1 (7.1) | 0 | 12 (85.7) |
| Cangola | 18 | 3 (16.7) | 1 (5.6) | 2 (11.1) | 0 | 2 (11.1) | 16 (88.9) |
| Damba | 22 | 9 (40.9) | 8 (36.4) | 4 (18.2) | 3 (13.6) | 1 (4.6) | 14 (63.6) |
| Maquela do Zombo | 21 | 14 (66.7) | 7 (33.3) | 0 | 0 | 0 | 15 (71.4) |
| Milunga | 10 | 5 (50.0) | 3 (30.0) | 1 (10.0) | 1 (10.0) | 0 | 9 (90.0) |
| Mucaba | 13 | 5 (38.5) | 2 (15.4) | 0 | 0 | 0 | 13 (100) |
| Negage | 18 | 10 (55.6) | 9 (50.0) | 7 (38.9) | 3 (16.7) | 4 (22.2) | 16 (88.9) |
| Puri | 10 | 8 (80.0) | 6 (60.0) | 0 | 0 | 0 | 9 (90.0) |
| Quimbele | 24 | 5 (20.8) | 4 (16.7) | 0 | 0 | 0 | 18 (75.0) |
| Quitexe | 6 | 2 (33.3) | 0 | 1 (16.7) | 1 (16.7) | 02 | 6 (100) |
| Sanza Pombo | 30 | 14 (46.7) | 10 (33.3) | 5 (16.7) | 2 (6.7) | 3 (10.) | 21 (70.0) |
| Songo | 14 | 9 (64.3) | 3 (21.4) | 3 (21.4) | 2 (14.3) | 1 (7.1) | 11 (78.6) |
| Uige | 20 | 15 (75.0) | 13 (65.0) | 11 (55.0) | 6 (30.0) | 5 (25.0) | 18 (90.0) |
| **Zaire province** | **56** | **40 (71.4)** | **12 (21.4)** | **8 (14.3)** | **2 (3.6)** | **6 (10.7)** | **51 (91.1)** |
| Cuimba | 12 | 7 (58.3) | 2 (16.7) | 1 (8.3) | 0 | 1 (8.3) | 12 (100) |
| Mbanza Congo | 13 | 12 (92.3) | 3 (23.1) | 1 (7.7) | 0 | 1 (7.7) | 12 (92.3) |
| Noqui | 9 | 6 (66.7) | 0 | 0 | 0 | 0 | 6 (66.7) |
| Nzeto | 5 | 4 (80.0) | 2 (40.0) | 2 (40.0) | 1 (20.0) | 1 (20.0) | 4 (80.0) |
| Soyo | 11 | 7 (63.6) | 4 (36.4) | 4 (36.4) | 1 (9.1) | 3 (27.3) | 11 (100) |
| Tomboco | 6 | 4 (66.7) | 1 (16.7) | 0 | 0 | 0 | 6 (100) |
| **Total** | **575** | **315 (54.8)** | **217 (37.7)** | **134 (23.3)** | **85 (14.8)** | **49 (8.5)** | **472 (81.9)** |

*N* = number of school survey responses. *n* = number of positive responses.

**Additional File 6.** Factors on multivariate analysis associated with schistosomiasis infection (considering RDT trace readings as negative).

|  | ***S. mansoni*** | | | ***S. haematobium*** | | | **Any schistosomiasis** | | |
| --- | --- | --- | --- | --- | --- | --- | --- | --- | --- |
|  | ***n*/*N*** | **a*OR* (95% *CI*)** | ***P*-value** | ***n*/*N*** | **a*OR* (95% *CI*)** | ***P*-value** | ***n*/*N*** | **a*OR* (95% *CI*)** | ***P*-value** |
| **Sex** |  |  |  |  |  |  |  |  |  |
| Male | 866/8,550 | NS | NS | 524/8,550 | 1 | - | 1,318/8,550 | 1 | - |
| Female | 859/8,543 | NS | NS | **714/8,543** | **1.49 (1.18–1.88)** | **0.001** | 1,471/8,543 | 1.15 (0.98–1.36) | 0.09 |
| **Age group (years)** |  |  |  |  |  |  |  |  |  |
| <11 | 606/6,348 | NS | NS | 368/6,348 | 1 |  | 9116,348 | 1 | - |
| 11-12 | 550/5,436 | NS | NS | 410/5,436 | 1.33 (0.96–1.84) | 0.09 | 914/5,436 | 1.17 (0.96–1.43) | 0.11 |
| >12 | 540/5,204 | NS | NS | **454/5,204** | **2.00 (1.48–2.69)** | **< 0.001** | **929/5,204** | **1.39 (1.11–1.74)** | **0.004** |
| **School setting** |  |  |  |  |  |  |  |  |  |
| Rural | 1,365/14,249 | NS | NS | 954/14,249 | 1 | - | 2,178/14,249 | 1 | - |
| Urban | 360/2,844 | NS | NS | **284/2,844** | **1.70 (1.22–2.38)** | **0.002** | **611/2,844** | **1.51 (1.13–2.04)** | **0.006** |
| **Ecological zone of school** |  |  |  |  |  |  |  |  |  |
| Northern coastal | 16/930 | 1 | - | 78/930 | 1 | - | 93/930 | 1 | - |
| Coffee area | **719/3,303** | **23.64 (8.02–69.69)** | **< 0.001** | **126/3,303** | **0.44 (0.21–0.93)** | **0.03** | **811/3,303** | **4.02 (1.88–8.57)** | **< 0.001** |
| Central highland | **990/12,860** | **5.20 (1.85–14.61)** | **0.002** | **1,034/12,860** | **0.30 (0.15–0.63)** | **0.001** | 1,885/12,860 | 0.86 (0.42–1.76) | 0.67 |
| **Ethnicity** |  |  |  |  |  |  |  |  |  |
| Umbundo | 761/7,620 | 1 | - | 927/7,620 | 1 | - | 1,557/7,620 | 1 | - |
| Kikongo | 876/7,937 | 0.67 (0.44–1.02) | 0.06 | **275/7,937** | **0.20 (0.14–0.29)** | **< 0.001** | **1,111/7,937** | **0.40 (0.29–0.56)** | **< 0.001** |
| Kimbundo | 88/1,536 | 0.77 (0.38–1.57) | 0.47 | **36/1,536** | **0.20 (0.14–0.31)** | **< 0.001** | **121/1,536** | **0.43 (0.26–0.74)** | **0.002** |
| **School dewormed 2013/2014** |  |  |  |  |  |  |  |  |  |
| No | 303/3,061 | NS | NS | 261/3,061 | NS | NS | 543/3,061 | NS | NS |
| Yes | 1,422/14,032 | NS | NS | 977/14,032 | NS | NS | 2,246/14,032 | NS | NS |
| **Latrines at school** |  |  |  |  |  |  |  |  |  |
| None/non-functional | 1,023/10,603 | NS | NS | 799/10,603 | NS | NS | 1,715/10,603 | NS | NS |
| Functional | 702/6,490 | NS | NS | 439/6,490 | NS | NS | 1,074/6,490 | NS | NS |
| **Water at school** |  |  |  |  |  |  |  |  |  |
| Not available | 1,270/13,063 | NS | NS | 888/13,063 | NS | NS | 2,028/13,063 | NS | NS |
| Available | 455/4,030 | NS | NS | 350/4,030 | NS | NS | 761/4,030 | NS | NS |

*n*/*N*: number of infections / at-risk population. a*OR*: adjusted odds ratio. *CI*: confidence interval. NS: did not meet significance threshold on univariate analysis (*P* < 0.2) to be included in multivariate analysis. Based on rapid diagnostic tests (trace readings considered negative) in determining schistosomiasis.
